# PD-1 signaling negatively regulates the common cytokine receptor γ chain via MARCH5-mediated ubiquitination and degradation to suppress anti-tumor immunity

**DOI:** 10.1038/s41422-023-00890-4

**Published:** 2023-11-06

**Authors:** Rui Liu, Lin-Wen Zeng, Hui-Fang Li, Jun-Ge Shi, Bo Zhong, Hong-Bing Shu, Shu Li

**Affiliations:** Department of Infectious Diseases, Zhongnan Hospital of Wuhan University; Frontier Science Center for Immunology and Metabolism; Medical Research Institute; Research Unit of Innate Immune and Inflammatory Diseases (2019RU063), Chinese Academy of Medical Sciences; Wuhan University, Wuhan, Hubei China

**Keywords:** Tumour immunology, Cell signalling

## Abstract

Combination therapy with PD-1 blockade and IL-2 substantially improves anti-tumor efficacy comparing to monotherapy. The underlying mechanisms responsible for the synergistic effects of the combination therapy remain enigmatic. Here we show that PD-1 ligation results in BATF-dependent transcriptional induction of the membrane-associated E3 ubiquitin ligase MARCH5, which mediates K27-linked polyubiquitination and lysosomal degradation of the common cytokine receptor γ chain (γ_c_). PD-1 ligation also activates SHP2, which dephosphorylates γ_c_^Y357^, leading to impairment of γ_c_ family cytokine-triggered signaling. Conversely, PD-1 blockade restores γ_c_ level and activity, thereby sensitizing CD8^+^ T cells to IL-2. We also identified Pitavastatin Calcium as an inhibitor of MARCH5, which combined with PD-1 blockade and IL-2 significantly improves the efficacy of anti-tumor immunotherapy in mice. Our findings uncover the mechanisms by which PD-1 signaling antagonizes γ_c_ family cytokine-triggered immune activation and demonstrate that the underlying mechanisms can be exploited for increased efficacy of combination immunotherapy of cancer.

## Introduction

The advances of cancer immunotherapy have revolutionized the treatment of cancer. Several types of immunotherapy, such as oncolytic viruses, cytokines, adoptive cell transfer and immune checkpoint inhibitors, have obtained durable clinical responses.^1–10^ At the forefront of clinical cancer immunotherapy is immune checkpoint blockade (ICB). Immune checkpoint molecules such as PD-1 of the coinhibitory signaling pathways are evolved to control the magnitude and duration of T cell responses to limit tissue damage and maintain self-tolerance under physiological and pathological conditions.^11^ However, tumor cells can hijack these inhibitory pathways to escape host immune surveillance such as by overexpressing the PD-1 ligand PD-L1.^11,12^ This provides the rationale for clinical application of immune checkpoint inhibitors in cancer immunotherapy. Clinically, antibodies blocking PD-1/PD-L1 axis reinvigorate the exhausted T cells in tumor microenvironment (TME) and show remarkable objective response and durable remission with acceptable toxicity profile in large numbers of tumors such as lymphoma and melanoma.^13,14^ However, not all patients respond to PD-1 monotherapy and there is considerable interest in developing combination therapy to improve the overall response rate and trigger more complete and durable response in patients with cancer.

Common cytokine receptor γ chain (γ_c_, also referred to as CD132) is a component of the receptors for interleukin-2 (IL-2), IL-4, IL-7, IL-9, IL-15 and IL-21.^15–17^ γ_c_ is constitutively expressed on various populations of immune cells; mutation of the gene encoding γ_c_ (*IL2RG*) results in X-linked severe combined immunodeficiency.^16,18,19^ Cytokines of the γ_c_ family exhibit pleiotropic functions in both innate and adaptive immune responses, contribute to development of T, B, NK and innate lymphoid cells (ILCs), promote either survival or death of immune cells depending on the context, and modulate differentiation of precursor immune cells into more terminally differentiated cells.^15,20,21^ Because of their important roles in regulating activity of T, NK and other immune cells, some of the γ_c_ family cytokines, such as IL-2, IL-9, IL-15 and IL-21, have shown strong anti-tumor effects.^15,20,21^ Studies of the γ_c_ family cytokines have allowed remarkable translational advances for autoimmune diseases as well as cancer.^22^

In the past years, combination immunotherapy of cancer has shown great promises.^23–25^ The immune checkpoint PD-1 blockade plus the γ_c_ family cytokine IL-2 is a promising combination of cancer immunotherapy with several clinical trials ongoing.^15,21,26^ IL-2 had been regarded as a therapeutic agent for cancer due to its powerful ability to stimulate proliferation of cytotoxic T lymphocytes and NK cells.^21,27^ However, only high-dose of IL-2 has shown therapeutic effects in certain cancer patients, and its widespread utilization is also limited by systemic toxicity,^27–29^ whereas combination therapy with PD-1 blockade and IL-2 is highly effective in cancer patients.^21,26^ Understanding the underlying mechanisms responsible for the synergistic effects of this combination is important to design better strategies for cancer immunotherapy.

In this study, we found that PD-1 signaling transcriptionally induced the E3 ubiquitin ligase MARCH5, which targeted γ_c_ for K27-linked polyubiquitination and lysosomal degradation. Additionally, PD-1 signaling also activated SHP2, which mediated dephosphorylation of γ_c_ and inhibition of signaling triggered by the γ_c_ family cytokines. These results suggest that PD-1 signaling antagonizes immune activation triggered by the γ_c_ family of cytokines through two distinct mechanisms. Consistently, PD-1 blockade restored the response of tumor-infiltrating CD8^+^ T cells to γ_c_ family cytokines. In addition, a MARCH5 inhibitor potently improved the efficacy of immunotherapy triggered by PD-1 blockade and IL-2 in mouse tumor models. Our results reveal the mechanisms by which PD-1 signaling inhibits γ_c_ family cytokine-triggered activation of CD8^+^ T cells and provide potential targets for increased efficacy of combination immunotherapy of cancer.

## Results

### PD-1 signaling down-regulates γ_c_ level

**Fig. 1:**
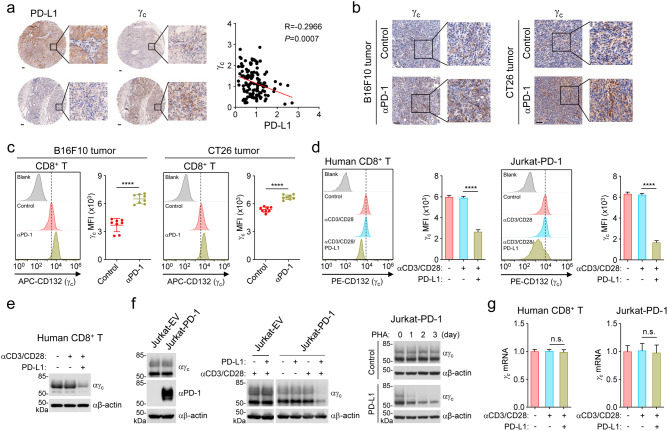
PD-1 signaling is negatively correlated with γ_c_ level. **a** γ_c_ level is negatively correlated with PD-L1 level in human NSCLC tumor biopsies. Representative images from IHC staining of γ_c_ and PD-L1 in human NSCLC tumor biopsies are shown (left panels). Quantification of PD-L1 and γ_c_ staining intensities was performed by semi-quantitative scoring (right panel). *n* = 127 independent samples, R = –0.2966. Correlation coefficients were calculated using the Pearson test. Two-sided *P*-value was given. Scale bars, 100 μm. **b** PD-1 blockade increases γ_c_ level in tumor tissues. B16F10 cells (5 × 10^5^) were subcutaneously injected into C57BL/6J mice. CT26 cells (5 × 10^5^) were subcutaneously injected into Balb/c mice. Mice were intraperitoneally injected with control or anti-PD-1 antibody (100 μg per mouse) every three days (four times in total) five days after inoculation of cells. After 17 days, tumor-bearing mice were euthanized and tumor tissues were analyzed. Representative images from IHC staining of γ_c_ in tumor sections are shown. Scale bars, 100 μm. **c** PD-1 blockade increases γ_c_ level in tumor-infiltrating CD8^+^ T cells. TILs were isolated from B16F10 tumor tissues (left panels) or CT26 tumor tissues (right panels), stained with the indicated antibodies and analyzed by flow cytometry. Graph shows mean ± SEM, *n* = 8 independent samples. Data were analyzed using Student’s unpaired *t*-test with GraphPad Prism 8. MFI, median fluorescence intensities. **d** PD-1 ligation down-regulates γ_c_ level. Human CD8^+^ T cells or PD-1-expressing Jurkat (Jurkat-PD-1) cells were stimulated with plate-bound anti-CD3 (1 μg/mL) and solubilized anti-CD28 (5 μg/mL) in the presence of plate-bound hPD-L1-Fc fusion protein or control hIgG1 (2 μg/mL) for 3 days. The cells were stained with the indicated antibodies and analyzed by flow cytometry. Graph shows mean ± SEM, *n* = 3 technical repeats. Data were analyzed using two-way ANOVA with GraphPad Prism 8. **e** The level of γ_c_ is down-regulated after PD-1 ligation. Human CD8^+^ T cells were stimulated with plate-bound anti-CD3 (1 μg/mL) and solubilized anti-CD28 (5 μg/mL) in the presence of plate-bound hPD-L1-Fc fusion protein or control hIgG1 (2 μg/mL) for 3 days before immunoblotting analysis with the indicated antibodies. **f** PD-1 ligation down-regulates γ_c_ level. Left panels: Jurkat cells transduced with empty vector (Jurkat-EV) or Jurkat-PD-1 cells were analyzed by immunoblots with the indicated antibodies. Middle panels: Jurkat-EV and Jurkat-PD-1 cells were stimulated with plate-bound anti-CD3 (1 μg/mL) and solubilized anti-CD28 (5 μg/mL) in the presence of plate-bound hPD-L1-Fc fusion protein or control hIgG1 (2 μg/mL) as indicated for 3 days before immunoblotting analysis with the indicated antibodies. Right panels: Jurkat-PD-1 cells were stimulated with PHA (50 ng/mL) in the presence of plate-bound hPD-L1-Fc fusion protein or control hIgG1 (2 μg/mL) for the indicated times before immunoblotting analysis with the indicated antibodies. **g** Effects of PD-1 ligation on transcription of γ_c_. Human CD8^+^ T cells or Jurkat-PD-1 cells were stimulated with plate-bound anti-CD3 (1 μg/mL) and solubilized anti-CD28 (5 μg/mL) in the presence of plate-bound hPD-L1-Fc fusion protein or control hIgG1 (2 μg/mL) for 3 days before qPCR analysis of mRNA levels of the indicated genes. Graph shows mean ± SEM, *n* = 3 independent samples from one representative experiment. Data were analyzed using two-way ANOVA with GraphPad Prism 8. The experiments in **c**–**g** were repeated for at least two times with similar results.

To explore whether there is a regulatory relationship between PD-1 signaling and the γ_c_ family cytokines, we firstly analyzed the correlation between PD-L1 and γ_c_ level in TME. Immunohistochemistry (IHC) staining showed that γ_c_ level was negatively correlated with PD-L1 level in human non-small cell lung cancer (NSCLC) biopsies (Fig. 1a). Next, we used a PD-1 antibody to disrupt the PD-1/PD-L1 signaling axis in mouse B16F10 and CT26 tumor models and then analyzed γ_c_ level in the tumor tissues. The results showed that PD-1 blockade up-regulated γ_c_ level in tumor tissues (Fig. 1b). Tumor-infiltrating CD8^+^ T cells is the main killer cells and rely on the γ_c_ family cytokines for proliferation and activation. Therefore, we analyzed γ_c_ level in tumor-infiltrating CD8^+^ T cells. The results indicated that the level of γ_c_ was increased in these cells after PD-1 blockade treatment (Fig. 1c), suggesting that PD-1 signaling negatively regulates γ_c_ level in tumor-infiltrating CD8^+^ T cells.

We next investigated whether PD-1 signaling regulates γ_c_ level in human T cells in vitro. Notably, the ability of PD-1 to exert its inhibitory effects requires simultaneous activation of T-cell receptor (TCR), as TCR proximal Src family kinase-mediated phosphorylation of immunoreceptor tyrosine-based switch motif (ITSM) of PD-1 is required for its recruitment of SHP2 and inhibitory function.^30–33^ Flow cytometry analysis indicated that γ_c_ level was not markedly changed after TCR activation by anti-CD3 and anti-CD28 antibodies, but was significantly down-regulated after PD-1 ligation by PD-L1 in both primary human CD8^+^ T cells and Jurkat T cells ectopically expressing PD-1 (Jurkat-PD-1) (Fig. 1d). Immunoblotting analysis further confirmed that PD-L1 treatment caused down-regulation of γ_c_ level in primary human CD8^+^ T cells (Fig. 1e). Jurkat cells do not express PD-1 and PD-L1 stimulation of Jurkat cells activated by anti-CD3 and anti-CD28 failed to induce γ_c_ down-regulation (Fig. 1f). However, PD-L1 stimulation caused down-regulation of γ_c_ in Jurkat-PD-1 cells (in which PD-1 is ectopically expressed) activated by anti-CD3 and anti-CD28. In these experiments, PD-L1 did not down-regulate γ_c_ in Jurkat-PD-1 cells not pretreated with anti-CD3 and anti-CD28, suggesting that TCR activation is important for PD-L1-triggered down-regulation of γ_c_ (Fig. 1f). Consistently, PD-L1 also triggered down-regulation of γ_c_ in Jurkat-PD-1 cells pretreated with phytohaemagglutinin (PHA) (Fig. 1f). These results further support that PD-1 ligation by PD-L1 causes down-regulation of γ_c_ in activated T cells. Quantitative real-time PCR (qPCR) experiments indicated that PD-L1 co-stimulation did not affect the mRNA level of γ_c_ in human primary CD8^+^ T cells or Jurkat-PD1 cells (Fig. 1g), suggesting that PD-1 signaling regulates γ_c_ at the protein but not mRNA level. Together, these results suggest that PD-1 signaling down-regulates γ_c_ level in human T cells.

### MARCH5 mediates K27-linked polyubiquitination and degradation of γ_c_

**Fig. 2:**
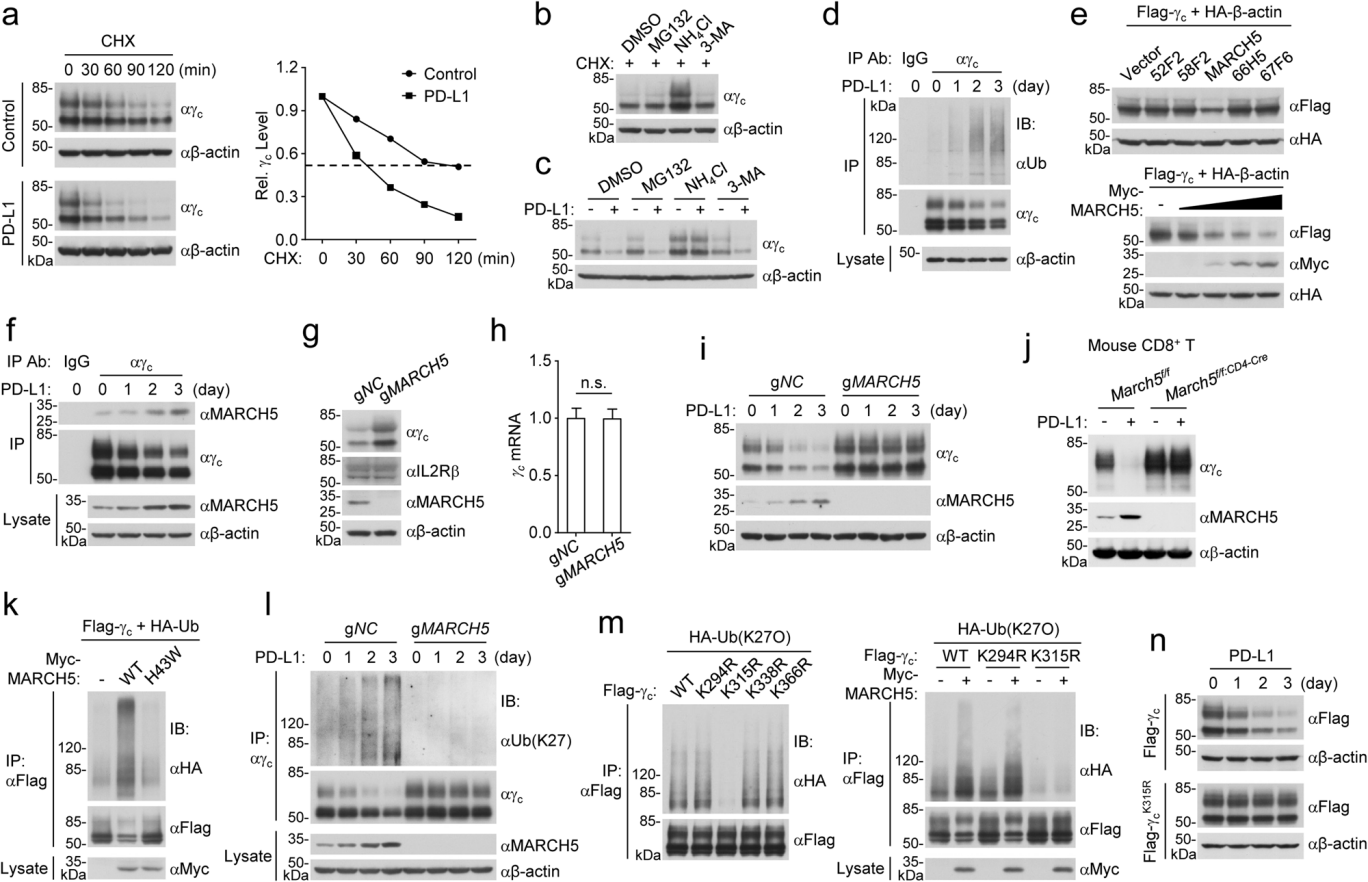
MARCH5 mediates K27-linked polyubiquitination and lysosomal degradation of γ_c_. **a** PD-1 ligation promotes γ_c_ degradation. Jurkat-PD-1 cells were pre-stimulated with PHA (50 ng/mL) for 36 h and then treated with CHX (0.1 mM) for the indicated times before immunoblotting analysis with the indicated antibodies. The γ_c_ band intensities relative to the corresponding β-actin bands were shown in the histograph. **b** NH_4_Cl inhibits γ_c_ degradation. Jurkat cells were pre-treated with MG132 (100 μM), NH_4_Cl (25 mM) or 3-MA (500 ng/mL) for 4 h, and then treated with CHX (0.1 mM) for 2 h before immunoblotting analysis with the indicated antibodies. **c** NH_4_Cl inhibits PD-1 ligation-induced degradation of γ_c_. Jurkat-PD-1 cells were stimulated with PHA (50 ng/mL) in the presence of plate-bound hPD-L1-Fc fusion protein or control hIgG1 (2 μg/mL) for 36 h, and then treated with MG132 (100 μM), NH_4_Cl (25 mM) or 3-MA (500 ng/mL) for 12 h before immunoblotting analysis with the indicated antibodies. **d** PD-1 ligation induces polyubiquitination of γ_c_. Jurkat-PD-1 cells were stimulated with PHA (50 ng/mL) in the presence of plate-bound hPD-L1-Fc fusion protein for the indicated times before co-immunoprecipitation and immunoblotting analysis with the indicated antibodies. **e** MARCH5 promotes degradation of γ_c_ in a dose-dependent manner. HEK293 cells were transfected with the indicated plasmids for 24 h before immunoblot analysis with the indicated antibodies. **f** Association of γ_c_ with MARCH5. Jurkat-PD-1 cells were stimulated with PHA (50 ng/mL) in the presence of plate-bound hPD-L1-Fc fusion protein for the indicated times before co-immunoprecipitation and immunoblotting analysis with the indicated antibodies. **g** MARCH5-deficiency up-regulates the level of γ_c_ but not IL2Rβ. Control or MARCH5-deficient Jurkat cells were collected for immunoblotting analysis with the indicated antibodies. **h** Effects of MARCH5-deficiency on transcription of γ_c_. Control or MARCH5-deficient Jurkat cells were analyzed by qPCR for mRNA levels of the indicated genes. Graph shows mean ± SEM, *n* = 3 independent samples from one representative experiment. Data were analyzed using two-way ANOVA with GraphPad Prism 8. **i** MARCH5-deficiency impairs PD-1 ligation-induced degradation of γ_c_. Control or MARCH5-deficient Jurkat-PD-1 cell were stimulated with PHA (50 ng/mL) in the presence of plate-bound hPD-L1-Fc fusion protein for the indicated times before immunoblotting analysis with the indicated antibodies. **j** PD-1 ligation-induced degradation of γ_c_ is impaired in MARCH5-deficient cells. Mouse CD8^+^ T cells from sex- and age-matched *March5*^*f/f*^ or *March5*^*f/f:CD4-Cre*^ mice were stimulated with plate-bound anti-CD3 (1 μg/mL) and solubilized anti-CD28 (5 μg/mL) in the presence of plate-bound mPD-L1-Fc fusion protein or control hIgG1 (2 μg/mL) for 3 days before immunoblotting analysis with the indicated antibodies. **k** MARCH5 promotes polyubiquitination of γ_c_. HEK293 cells were transfected with the indicated plasmids for 24 h before co-immunoprecipitation and immunoblotting analysis with the indicated antibodies. **l** MARCH5-deficiency impairs PD-L1-induced K27-linked polyubiquitination of γ_c_. Control or MARCH5-deficient PD-1-expressing Jurkat cells were stimulated with PHA (50 ng/mL) in the presence of plate-bound hPD-L1-Fc fusion protein for the indicated times before co-immunoprecipitation and immunoblotting analysis with the indicated antibodies. **m** MARCH5 increases K27-linked polyubiquitination of wild-type γ_c_ and γ_c_^K294R^ but not γ_c_^K315R^. HEK293 cells were transfected with the indicated plasmids for 24 h before co-immunoprecipitation and immunoblotting analysis with the indicated antibodies. **n** PD-1 induces down-regulation of wild-type γ_c_ but not γ_c_^K315R^. Jurkat-PD-1 cells were expressed with Flag-tagged wild-type γ_c_ or γ_c_^K315R^ mutant and then stimulated with PHA (50 ng/mL) in the presence of plate-bound hPD-L1-Fc fusion protein for the indicated times before immunoblotting analysis with the indicated antibodies. All the experiments were repeated for at least two times with similar results.

We next investigated the mechanisms responsible for PD-1 signaling-triggered down-regulation of γ_c_. The protein synthesis inhibitor cycloheximide (CHX) treatment showed that γ_c_ had a shorter half-life after PD-L1 stimulation in Jurkat-PD-1 cells (Fig. 2a), suggesting that PD-1 ligation promotes γ_c_ degradation. γ_c_ degradation after termination of protein synthesis by CHX was markedly inhibited by the lysosomal inhibitor ammonium chloride (NH_4_Cl) but not the proteasome inhibitor MG132 or autophagosome inhibitor 3-methyladenine (3-MA) in Jurkat cells (Fig. 2b). PD-L1-induced degradation of γ_c_ was also inhibited by NH_4_Cl but not MG132 or 3-MA in Jurkat-PD-1 cells (Fig. 2c). Together, these results suggest that PD-1 signaling results in γ_c_ degradation through the lysosomal route.

It has been demonstrated that polyubiquitination of proteins target them for degradation by various routes including the lysosomal pathway.^34^ Consistently, we found that PD-L1 induced polyubiquitination and down-regulation of γ_c_ in Jurkat-PD1 cells, which was most dramatic at 2 days after PD-L1 stimulation (Fig. 2d). We next attempted to identify the E3 ubiquitin ligases that catalyze degradation of γ_c_. We screened 196 ubiquitin-related proteins for their abilities to regulate γ_c_ level by co-transfection experiments in HEK293 cells. These efforts led to the identification of MARCH5, which caused down-regulation of γ_c_ in a dose-dependent manner (Fig. 2e) but had no marked effects on the γ_c_ family cytokine receptor IL2RB, IL4R or IL7R (Supplementary information, Fig. S1a). There are 11 members in the MARCH E3 ligase family.^35^ Overexpression of MARCH5 but not other 10 MARCH family members markedly down-regulated the level of γ_c_ (Supplementary information, Fig. S1b). These results suggest that MARCH5 specifically down-regulates γ_c_.

Previous studies showed that MARCH5 is a membrane-embedded E3 ligase that is known to be present on the mitochondrial outer membrane or peroxisomal membrane.^36,37^ Since γ_c_ is a plasma membrane receptor which is synthesized and translocated from the ER-Golgi-endosome system,^38^ we next investigated whether MARCH5 can colocalize with γ_c_. Confocal microscopy experiments showed that γ_c_ was partially colocalized with MARCH5 at the plasma membrane, endosomes and lysosomes but not at the mitochondria, ER, Golgi and peroxisomes (Supplementary information, Fig. S1c, d). Consistent with the previous reports,^36,37^ MARCH5 was also partially located at the mitochondria and peroxisomes (Supplementary information, Fig. S1d). In addition, endogenous co-immunoprecipitation experiments indicated that γ_c_ was basally associated with MARCH5 and PD-L1 stimulation promoted their association in Jurkat-PD-1 cells (Fig. 2f). These results support the conclusion that γ_c_ is polyubiquitinated by MARCH5 and degraded by the lysosomal pathway. Interestingly, these experiments also indicated that MARCH5 was induced after PD-L1 stimulation, which reached to the highest level 3 days after PD-L1 stimulation, and the induction of MARCH5 was correlated with down-regulation of γ_c_ (Fig. 2f).

We generated MARCH5-deficient Jurkat cells using the CRISPR-Cas9 method and found that MARCH5-deficiency up-regulated the protein level of γ_c_ but not IL2Rβ (Fig. 2g). MARCH5-deficiency did not affect the mRNA level of γ_c_ in Jurkat cells (Fig. 2h). In addition, MARCH5-deficiency increased γ_c_ level in human T lymphoid leukemia HPB-ALL cells (Supplementary information, Fig. S2a) and murine T lymphocyte CTLL2 cells (Supplementary information, Fig. S2b). Furthermore, we generated *March5* T cell-specific knockout mice (*March5*^*f/f:CD4-Cre*^) and isolated the thymocytes from *March5*^*f/f*^ or *March5*^*f/f:CD4-Cre*^ mice. The results showed that MARCH5-deficiency increased γ_c_ level in thymocytes (Supplementary information, Fig. S2c). Previously, it has been demonstrated that phosphorylation of STAT5^Y694/Y699^ is a hallmark of γ_c_ family cytokine-triggered signaling activation.^15^ Consistently, MARCH5-deficiency increased the γ_c_ family cytokine IL-7- and IL-9-induced phosphorylation of STAT5^Y694/Y699^ in HPB-ALL cells, which has been shown to be responsive to the two cytokines (Supplementary information, Fig. S2a),^39^ whereas MARCH5-deficiency increased IL-2-induced phosphorylation of STAT5^Y694/Y699^ in CTLL2 cells which was responsive to IL-2 (Supplementary information, Fig. S2b).^40^ In addition, Il-7-induced phosphorylation of STAT5^Y694/Y699^ was increased in March5-deficient thymocytes in comparison to their wild-type counterparts (Supplementary information, Fig. S2c). These experiments also showed that γ_c_ levels were not markedly changed following stimulation by the γ_c_ family cytokines IL-2, IL-7 and IL-9 (Supplementary information, Fig. S2a–c). In addition, NH_4_Cl treatment inhibited MARCH5-mediated degradation of γ_c_ in HEK293 cells (Supplementary information, Fig. S2d). Moreover, MARCH5-deficiency impaired PD-L1-induced γ_c_ degradation in Jurkat-PD-1 cells (Fig. 2i) and mouse CD8^+^ T cells (Fig. 2j). Taken together, these results suggest that MARCH5 mediates PD-L1-induced lysosomal degradation of γ_c_ and inhibition of the γ_c_ family cytokine-triggered signaling in various cells.

We next investigated the molecular mechanisms of MARCH5-mediated polyubiquitination of γ_c_. Overexpression of MARCH5 but not its inactive mutant MARCH5^H43W^ promoted polyubiquitination of γ_c_ in HEK293 cells (Fig. 2k). Utilizing ubiquitin mutants in which one or six lysine residues are replaced with arginine (R), we found that MARCH5 increased K27- but not other lysine residue-linked polyubiquitination of γ_c_ (Supplementary information, Fig. S3a). Endogenous ubiquitination assays indicated that PD-L1 stimulation increased K27-linked polyubiquitination and degradation of γ_c_, and these effects were impaired in MARCH5-deficient Jurkat-PD-1 cells (Fig. 2l). Collectively, these results suggest that MARCH5 mediates K27-linked polyubiquitination and degradation of γ_c_ following PD-1 ligation.

To further identify the residues in γ_c_ that are conjugated with K27-linked polyubiquitin chains by MARCH5, we individually mutated each of the 4 lysine residues within the intracellular region (284–369 aa) of γ_c_, K294, K315, K338 and K363, to arginine and examined whether these mutants could be modified by K27-linked polyubiquitination. The results indicated that mutation of K315 but not the other 3 lysine residues in γ_c_ to arginine dramatically reduced its K27-linked polyubiquitination, and MARCH5 increased K27-linked polyubiquitination and down-regulation of wild-type γ_c_ and γ_c_^K294R^ but not γ_c_^K315R^ (Fig. 2m). Consistently, PD-L1 stimulation induced down-regulation of wild-type γ_c_ but not γ_c_^K315R^ in Jurkat-PD-1 cells (Fig. 2n). Reconstitution of γ_c_^K315R^ in γ_c_-deficient HPB-ALL cells increased IL-7- and IL-9-induced phosphorylation of STAT5^Y694/Y699^ in comparison to cells reconstituted with wild-type γ_c_ (Supplementary information, Fig. S3b). In these reconstitution experiments, the mRNA levels of γ_c_^K315R^ and wild-type γ_c_ were comparable, but the protein level of γ_c_^K315R^ was dramatically up-regulated compared to its wild-type counterparts (Supplementary information, Fig. S3b). Taken together, these results suggest that MARCH5 targets γ_c_^K315^ for its K27-linked polyubiquitination and degradation.

### USP5 antagonizes MARCH5-mediated γ_c_ polyubiquitination and degradation

**Fig. 3:**
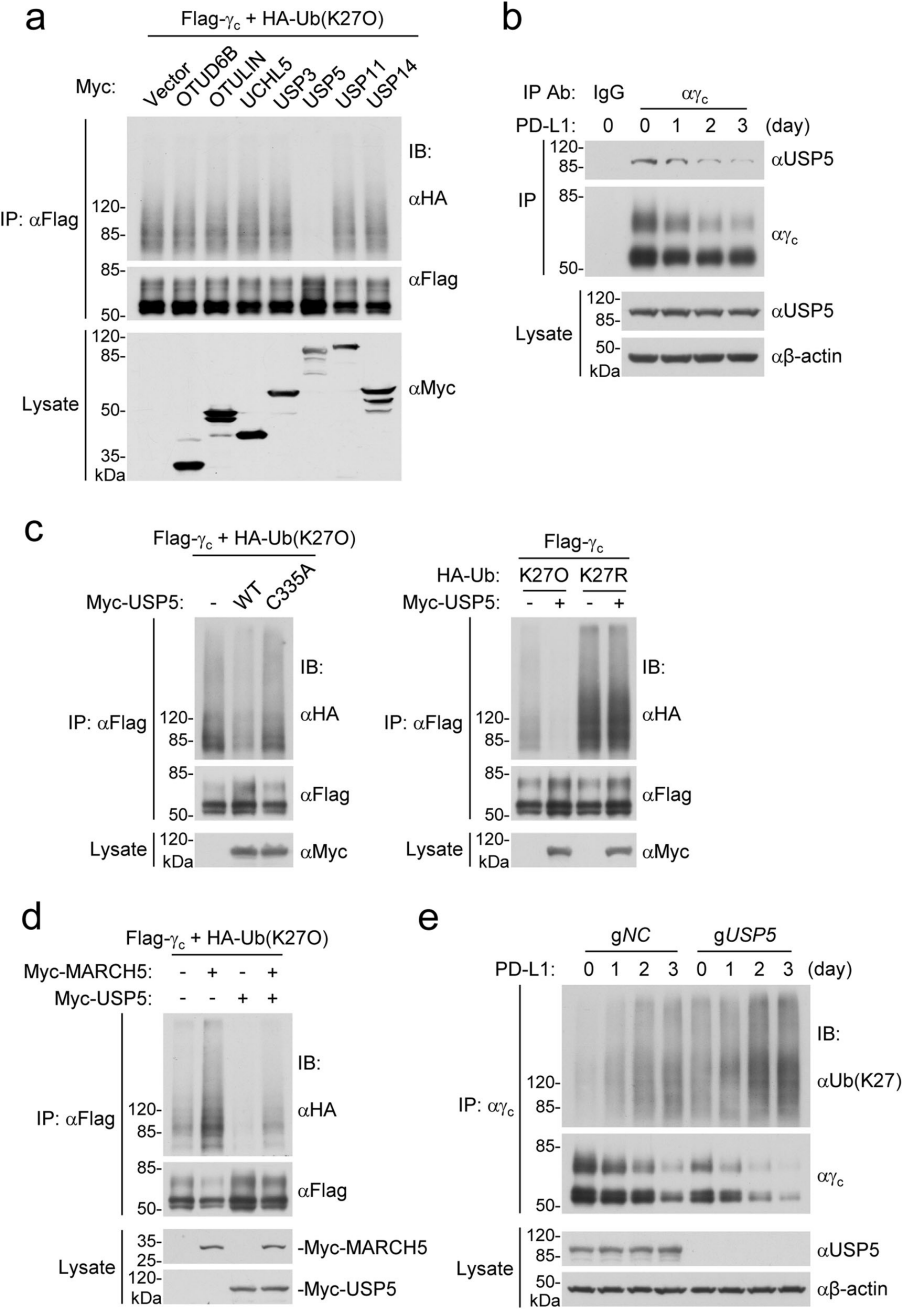
USP5 removes K27-linked polyubiquitin moieties from γ_c_. **a** USP5 removes K27-linked polyubiquitin moieties from γ_c_. HEK293 cells were transfected with the indicated plasmids for 24 h before immunoblotting analysis with the indicated antibodies. **b** Association of γ_c_ with USP5. Jurkat-PD-1 cells were stimulated with PHA (50 ng/mL) in the presence of plate-bound hPD-L1-Fc fusion protein for the indicated times before co-immunoprecipitation and immunoblotting analysis with the indicated antibodies. **c** USP5 but not its enzymatic inactive mutant USP5^C335A^ removes K27-linked polyubiquitin moieties from γ_c_. HEK293 cells were transfected with the indicated plasmids for 24 h before co-immunoprecipitation and immunoblotting analysis with the indicated antibodies. **d** USP5 removes K27-linked polyubiquitin moieties of γ_c_ catalyzed by MARCH5. HEK293 cells were transfected with the indicated plasmids for 24 h before immunoblotting analysis with the indicated antibodies. **e** USP5-deficiency enhances PD-1 ligation-induced K27-linked polyubiquitination of γ_c_. Control or USP5-deficient Jurkat-PD-1 cells were stimulated with PHA (50 ng/mL) in the presence of plate-bound hPD-L1-Fc fusion protein for the indicated times before co-immunoprecipitation and immunoblotting analysis with the indicated antibodies. All the experiments were repeated for at least two times with similar results.

We next attempted to identify deubiquitinate enzymes that are responsible for removing K27-linked polyubiquitin moieties conjugated to γ_c_. γ_c_-bound proteins were immunoprecipitated with anti-γ_c_ and analyzed by mass spectrometry. Among the 184 proteins identified, 7 are deubiquitinate enzymes (Supplementary information, Table S1). Co-transfection experiments indicated that only USP5 but not the other 6 enzymes removed K27-linked polyubiquitin moieties from γ_c_ in HEK293 cells (Fig. 3a). Endogenous co-immunoprecipitation experiments indicated that γ_c_ was constitutively associated with USP5 in Jurkat-PD-1 cells (Fig. 3b). USP5 but not its enzymatic inactive mutant USP5^C335A^ removed K27-linked polyubiquitin moieties from γ_c_ and up-regulated the level of γ_c_ in HEK293 cells (Fig. 3c). In addition, USP5 removed K27-linked polyubiquitination of γ_c_ catalyzed by MARCH5 (Fig. 3d). In these experiments, it is notable that overexpression of USP5 up-regulated whereas MARCH5 down-regulated the levels of γ_c_ (Fig. 3c, d). Endogenous ubiquitination assays indicated that PD-L1 stimulation induced K27-linked polyubiquitination of γ_c_, which was increased in USP5-deficient Jurkat-PD-1 cells (Fig. 3e). In these experiments, the protein level of γ_c_ in USP5-deficient cells was also dramatically down-regulated compared with those in wild-type cells (Fig. 3e). USP5-deficiency also down-regulated the level of γ_c_ in HPB-ALL and CTLL2 cells, and inhibited IL-2-, IL-7- and IL-9-induced phosphorylation of STAT5 at Y694/Y699 in these cells (Supplementary information, Fig. S4). These data suggest that USP5 positively regulates γ_c_ level as well as the γ_c_ family cytokine-triggered signaling in various cells.

### PD-1 signaling promotes γ_c_ degradation by inducing MARCH5 transcription

**Fig. 4:**
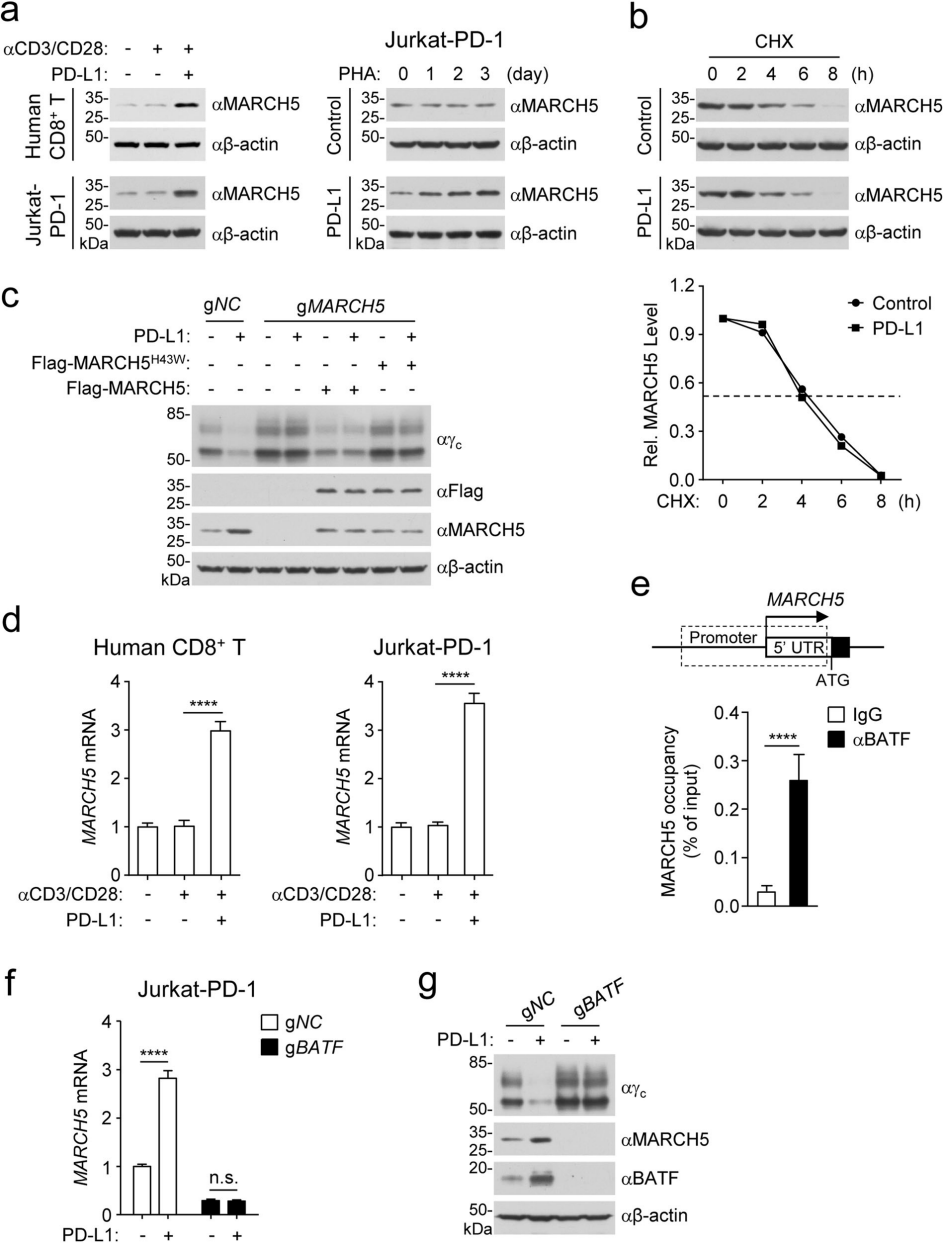
PD-1 signaling promotes γ_c_ degradation by inducing transcription of MARCH5. **a** MARCH5 is up-regulated after PD-L1 but not PHA stimulation. Human CD8^+^ T cells or Jurkat-PD-1 cells were stimulated with plate-bound anti-CD3 (1 μg/mL) and solubilized anti-CD28 (5 μg/mL) in the presence of plate-bound hPD-L1-Fc fusion protein or control hIgG1 (2 μg/mL) for 3 days before immunoblotting analysis with the indicated antibodies (left panels). Jurkat-PD-1 cells were stimulated with PHA (50 ng/mL) in the presence of plate-bound hPD-L1-Fc fusion protein or control hIgG1 (2 μg/mL) for the indicated times before immunoblotting analysis with the indicated antibodies (right panels). **b** Effects of PD-1 ligation on MARCH5 degradation. Jurkat-PD-1 cells were pre-stimulated with PHA (50 ng/mL) in the presence of plate-bound hPD-L1-Fc fusion protein or control hIgG1 (2 μg/mL) for 36 h and then treated with CHX (0.1 mM) for the indicated times before immunoblotting analysis with the indicated antibodies. The MARCH5 band intensities relative to the corresponding β-actin bands were shown in the histograph. **c** MARCH5-deficiency impairs PD-1 ligation-induced degradation of γ_c_. Control or MARCH5-deficient Jurkat-PD-1 cells were reconstituted with wild-type MARCH5 or MARCH5^H43W^ mutant and then stimulated with PHA (50 ng/mL) in the presence of plate-bound hPD-L1-Fc fusion protein or control hIgG1 (2 μg/mL) for 2 days before immunoblotting analysis with the indicated antibodies. **d** Effects of PD-1 ligation on MARCH5 mRNA level. Human CD8^+^ T cells or Jurkat-PD-1 cells were stimulated with plate-bound anti-CD3 (1 μg/mL) and solubilized anti-CD28 (5 μg/mL) in the presence of plate-bound hPD-L1-Fc fusion protein or control hIgG1 (2 μg/mL) for 3 days before qPCR analysis of mRNA levels of the indicated genes. Graph shows mean ± SEM, *n* = 3 independent samples from one representative experiment. Data were analyzed using two-way ANOVA with GraphPad Prism 8. **e** BATF binds to the promoter region of *MARCH5* gene. Jurkat-PD-1 cells were analyzed by ChIP with the indicated antibodies, and then de-crosslinked DNA was subjected to qPCR analysis using specific primers. Graph shows mean ± SEM, *n* = 3 independent samples from one representative experiment. Data were analyzed using a Student’s unpaired *t*-test with GraphPad Prism 8. **f** BATF-deficiency impairs PD-1 ligation-induced transcription of MARCH5. Control or BATF-deficient Jurkat-PD-1 cells were stimulated with PHA (50 ng/mL) in the presence of plate-bound hPD-L1-Fc fusion protein or control hIgG1 (2 μg/mL) for 2 days before qPCR analysis of mRNA levels of the indicated genes. Graph shows mean ± SEM, *n* = 3 independent samples from one representative experiment. Data were analyzed using two-way ANOVA with GraphPad Prism 8. **g** BATF-deficiency impairs PD-1 ligation-induced degradation of γ_c_. Control or BATF-deficient Jurkat-PD-1 cells were stimulated with PHA (50 ng/mL) in the presence of plate-bound hPD-L1-Fc fusion protein or control hIgG1 (2 μg/mL) for 2 days before immunoblotting analysis with the indicated antibodies. All the experiments were repeated for at least two times with similar results.

In our experiments, we found that PD-1 ligation had no marked effects on the protein level of USP5 as well as its association with γ_c_ (Fig. 3b). USP5-deficiency down-regulated the basal level of γ_c_ but did not affect PD-L1-induced γ_c_ degradation (Fig. 3e). These results suggest that USP5 is not required for PD-1 ligation-induced γ_c_ degradation. On the other hand, we found that the protein level of MARCH5 was not markedly changed after TCR activation, but was up-regulated following PD-L1 stimulation in human CD8^+^ T cells or Jurkat-PD-1 cells (Figs. 4a and 2f, i, j, l). PD-L1 stimulation did not affect the half-life of MARCH5 in CHX-treated Jurkat-PD-1 cells (Fig. 4b), suggesting that PD-1 ligation does not affect its stability. MARCH5-deficiency caused up-regulation of γ_c_ protein level, which was reversed by reconstitution with wild-type MARCH5 but not MARCH5^H43W^ in Jurkat-PD-1 cells. Additionally, PD-1 ligation down-regulated γ_c_ level in wild-type but not MARCH5-deficient cells ectopically reconstituted with wild-type MARCH5 in Jurkat-PD-1 cells (Fig. 4c). qPCR analysis indicated that PD-L1 treatment up-regulated *MARCH5* mRNA level in human CD8^+^ T and Jurkat-PD-1 cells (Fig. 4d). These results suggest that PD-1 ligation regulates MARCH5 level at mRNA but not protein level.

It has been demonstrated that PD-1 ligation induces expression of certain transcription factors including the AP-1 family member BATF, which inhibits T cell function.^13,41^ Analysis of the Gene Transcription Regulation Database (GTRD, https://gtrd.biouml.org/) indicates that BATF can bind to the promoter region of *MARCH5* gene. Consistently, chromatin immunoprecipitation (ChIP) experiments confirmed that BATF bound to the promoter region of *MARCH5* gene in Jurkat-PD-1 cells (Fig. 4e). BATF-deficiency caused down-regulation and impairment of PD-L1-induced up-regulation of *MARCH5* mRNA level in Jurkat-PD-1 cells (Fig. 4f). PD-1 ligation up-regulated the level of BATF, and BATF-deficiency up-regulated γ_c_ level and inhibited PD-L1-induced down-regulation of γ_c_ level in Jurkat-PD-1 cells (Fig. 4g). These results suggest that the basal level of γ_c_ is constitutively regulated by basal BATF and PD-1 signaling transcriptionally induces MARCH5 in a BATF-dependent manner.

### PD-1-triggered SHP2 activation induces dephosphorylation of γ_c_^Y357^

**Fig. 5:**
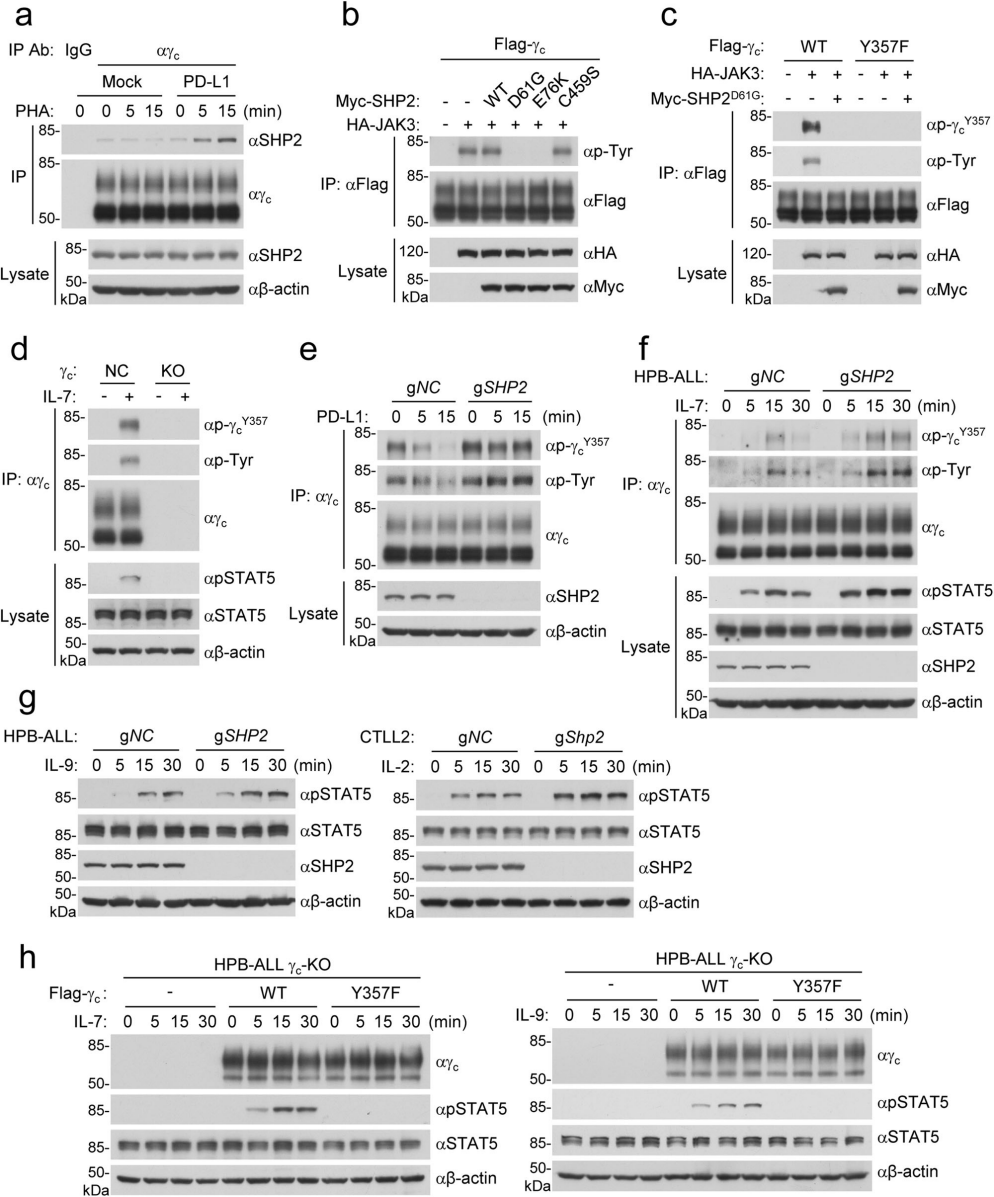
PD-1 signaling activates SHP2 to induce dephosphorylation of γ_c_^Y357^. **a** Association of γ_c_ with SHP2. Jurkat-PD-1 cells were stimulated with PHA (150 ng/mL) in the presence of plate-bound hPD-L1-Fc fusion protein or control hIgG1 (2 μg/mL) for the indicated times before co-immunoprecipitation and immunoblotting analysis with the indicated antibodies. **b** Effects of SHP2 on dephosphorylation of γ_c_. HEK293 cells were transfected with the indicated plasmids for 24 h before co-immunoprecipitation and immunoblotting analysis with the indicated antibodies. **c** JAK3 mediates phosphorylation of γ_c_^Y357^. HEK293 cells were transfected with the indicated plasmids for 24 h before co-immunoprecipitation and immunoblotting analysis with the indicated antibodies. **d** IL-7 treatment induces γ_c_^Y357^ phosphorylation. Control or γ_c_-deficient HPB-ALL cells were stimulated with IL-7 (100 ng/mL) for the indicated times before co-immunoprecipitation and immunoblotting analysis with the indicated antibodies. **e** SHP2-deficiency impairs PD-L1-induced γ_c_^Y357^ dephosphorylation. Control or SHP2-deficient Jurkat-PD-1 cells were stimulated with PHA (150 ng/mL) in the presence of plate-bound hPD-L1-Fc fusion protein for the indicated times before co-immunoprecipitation and immunoblotting analysis with the indicated antibodies. **f** SHP2-deficiency enhances IL-7-induced γ_c_^Y357^ phosphorylation. Control or SHP2-deficient HPB-ALL cells were stimulated with IL-7 (100 ng/mL) for the indicated times before co-immunoprecipitation and immunoblotting analysis with the indicated antibodies. **g** SHP2-deficiency promotes γ_c_ family cytokine-induced phosphorylation of STAT5^Y694/Y699^. Control or SHP2-deficient HPB-ALL cells were stimulated with IL-9 (100 ng/mL) for the indicated times before immunoblotting analysis with the indicated antibodies (left panels). Control or SHP2-deficient CTLL2 cells were stimulated with IL-2 (400 IU/mL) for the indicated times before immunoblotting analysis with the indicated antibodies (right panels). **h** Effects of γ_c_^Y357F^ mutant on the γ_c_ family cytokine-induced phosphorylation of STAT5 ^Y694/Y699^. γ_c_-deficient HPB-ALL cells were reconstituted with wild-type γ_c_ or γ_c_^Y357F^ mutant and then stimulated with IL-7 (100 ng/mL) or IL-9 (100 ng/mL) for the indicated time lengths before immunoblotting analysis with the indicated antibodies. All the experiments were repeated for at least two times with similar results.

It has been shown that the allosteric dephosphorylating enzyme SHP2 is recruited to and activated in the complex of PD-1 upon PD-1 ligation, and the activated SHP2 mediates dephosphorylation of the TCR and CD28 proximal signaling molecules which leads to suppression of T cells.^13,31,33^ Our biochemical purification and mass spectrometry analysis indicated that SHP2 was a potential γ_c_-bound proteins (Supplementary information, Table S1). Endogenous co-immunoprecipitation experiments indicated that γ_c_ was weakly associated with SHP2 and PD-L1 stimulation promoted their association at 5 min post stimulation in Jurkat-PD-1 cells (Fig. 5a). These results suggest that PD-1 ligation promotes a physical association of SHP2 with γ_c_.

We next investigated whether SHP2 mediates dephosphorylation of γ_c_. It has been demonstrated that the tyrosine kinase JAK3 mediates phosphorylation of γ_c_ upon stimulation of the γ_c_ family cytokines. Overexpression of the SHP2 gain-of-function mutant D61A or E76K but not the wild-type SHP2 or dominant negative SHP2 mutant C459S abolished JAK3-mediated phosphorylation of γ_c_ (Fig. 5b). In similar experiments, SHP2 was not associated with JAK3 and overexpression of the SHP2 gain-of-function mutant D61A or E76K did not abolish the phosphorylation of JAK3 (Supplementary information, Fig. S5a), suggesting that SHP2 does not target JAK3. The level of JAK3 was not markedly changed in SHP2-deficient HPB-ALL cells comparing to their wild-type counterparts (Supplementary information, Fig. S5b), There are 4 tyrosine residues in the intracellular region (284–369 aa) of γ_c_ (Supplementary information, Fig. S5c). Mutagenesis indicated that mutation of Y357 but not the other 3 tyrosine residues to phenylalanine impaired JAK3-mediated tyrosine phosphorylation of γ_c_ (Supplementary information, Fig. S5d). Sequence analysis indicated that γ_c_^Y357^ was conserved in various vertebrate species (Supplementary information, Fig. S5c). To determine whether SHP2 dephosphorylates γ_c_^Y357^, we generated a rabbit polyclonal antibody specific for Y357-phosphorylated γ_c_ (p-γ_c_^Y357^). Immunoblotting analysis confirmed that γ_c_^Y357^ was phosphorylated following co-expression of JAK3, which was reversed by the gain-of-function mutant SHP2^D61G^ in HEK293 cells (Fig. 5c). Endogenous γ_c_^Y357^ was phosphorylated following IL-7 stimulation, which was not seen in un-stimulated or γ_c_-deficient HPB-ALL cells (Fig. 5d). Since Jurkat is a γ_c_-dependent cell line in which γ_c_-mediated signaling is constitutively activated, γ_c_^Y357^ was constitutively phosphorylated and PD-L1 stimulation caused dephosphorylation of γ_c_^Y357^ as early as 5 min post stimulation, while SHP2-deficiency abolished PD-L1-induced dephosphorylation of γ_c_^Y357^ in Jurkat-PD-1 cells (Fig. 5e). SHP2-deficiency also enhanced IL-7-induced phosphorylation of γ_c_^Y357^ (Fig. 5f) as well as IL-2-, IL-7- and IL-9-induced phosphorylation of STAT5^Y694/Y699^ in HPB-ALL and CTLL2 cells, respectively (Fig. 5f, g), suggesting that SHP2 negatively regulates γ_c_ family cytokine-triggered signaling. In addition, the level of γ_c_ was not markedly changed in SHP2-deficient HPB-ALL or Jurkat-PD-1 cells comparing to their wild-type counterparts (Fig. 5e, f), suggesting that JAK3-mediated phosphorylation and SHP2-mediated dephosphorylation of γ_c_ is not involved in MARCH5-mediated down-regulation of γ_c_. Reconstitution of wild-type γ_c_ but not γ_c_^Y357F^ in γ_c_-deficient HPB-ALL cells restored IL-7- and IL-9-induced phosphorylation of STAT5^Y694/Y699^ (Fig. 5h). These results suggest that the phosphorylation of γ_c_^Y357^ is required for γ_c_ family cytokine-triggered signaling activation, while PD-1 ligation triggers SHP2-mediated dephosphorylation and inactivation of γ_c_.

### MARCH5 knockdown improves anti-tumor immunity and suppresses tumor growth

**Fig. 6:**
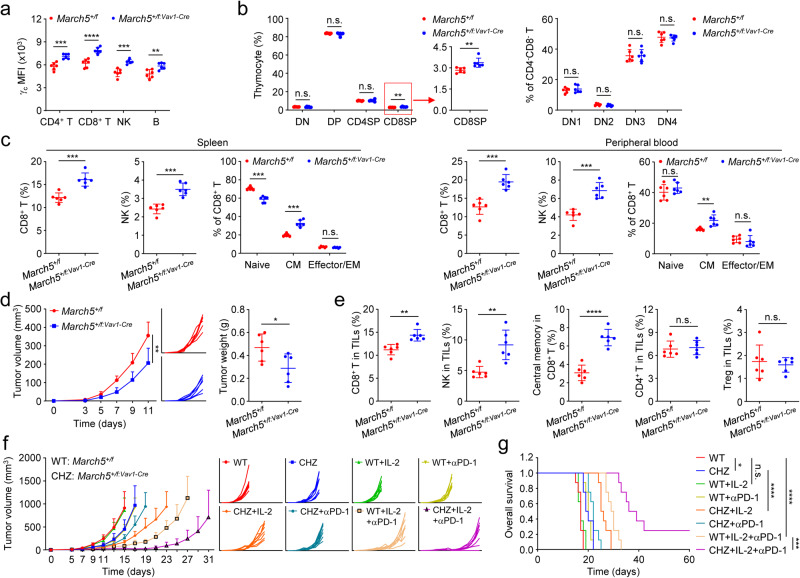
MARCH5 knockdown improves anti-tumor immunity and suppresses tumor growth. **a** Effects of MARCH5 knockdown on the level of γ_c_ in lymphocytes from spleen. Splenocytes from sex- and age-matched *March5*^*+/f*^ or *March5*^*+/f:Vav1-Cre*^ mice were stained with the indicated antibodies and analyzed by flow cytometry. Graph shows mean ± SEM, *n* = 6 independent samples. Data were analyzed using Student’s unpaired *t*-test with GraphPad Prism 8. MFI, median fluorescence intensities. **b** March5 knockdown increases the percentage of CD8^+^ single-positive (CD8SP) cells in thymocytes. Thymocytes from sex- and age-matched *March5*^*+/f*^ or *March5*^*+/f:Vav1-Cre*^ mice were analyzed by flow cytometry for the percentage of CD4^-^CD8^-^ double-negative (DN), CD4^+^CD8^+^ double-positive (DP), CD4^+^ single-positive (CD4SP) and CD8^+^ single-positive (CD8SP). The CD4^-^CD8^-^ double-negative (DN) cells were analyzed by flow cytometry for the percentage of CD44^+^ single-positive (DN1), CD44^+^CD25^+^ double-positive (DN2), CD25^+^ single-positive (DN3) and CD44^-^CD25^-^ double-negative (DN4).Graph shows mean ± SEM, *n* = 6 independent samples. Data were analyzed using Student’s unpaired *t*-test with GraphPad Prism 8. **c** Effects of MARCH5 knockdown on the percentages of CD8^+^ T and NK cells from spleen and the peripheral blood. Splenocytes or peripheral blood leukocytes from sex- and age-matched *March5*^*+/f*^ or *March5*^*+/f:Vav1-Cre*^ mice were analyzed by flow cytometry for the percentage of CD8^+^ T (CD3^+^CD8^+^), NK (CD3^-^NKp46^+^), native CD8^+^ T (CD44^low^CD62L^high^ CD8^+^ T cells), central memory CD8^+^ T (CD44^high^CD62L^high^ CD8^+^ T cells, CM) and effector/effector memory CD8^+^ T (CD44^high^CD62L^low^ CD8^+^ T cells, Effector/EM) cells. Graph shows mean ± SEM, *n* = 6 independent samples. Data were analyzed using Student’s unpaired *t*-test with GraphPad Prism 8. **d** MARCH5 knockdown inhibits tumor growth. Sex- and age-matched *March5*^*+/f*^ or *March5*^*+/f:Vav1-Cre*^ mice were subcutaneously injected with MC38 cells (5 × 10^5^). On day 3 after tumor cell inoculation, tumor sizes were measured every two days by caliper. Tumor-bearing mice were euthanized on day 13, and then tumor tissues were separated from the mice. Tumor weights were measured by Analytical Balance. Graph shows mean ± SEM, *n* = 6. Data were analyzed using Student’s unpaired *t*-test with GraphPad Prism 8. **e** MARCH5 knockdown increases the percentages of CD8^+^ T and NK cells in TILs. TILs were isolated from the MC38 tumor tissues in **d**. TILs were stained with the indicated antibodies and analyzed by flow cytometry. Graph shows mean ± SEM, *n* = 6 independent samples. Data were analyzed using Student’s unpaired *t*-test with GraphPad Prism 8. **f** Combination of IL-2 and PD-1 blockade has increased anti-tumor efficacy in *March5*^*+/f:Vav1-Cre*^ mice. *March5*^*+/f*^ and *March5*^*+/f:Vav1-Cre*^ mice were subcutaneously injected with MC38 cells (5 × 10^5^). On day 5 after tumor cell inoculation, mice were intraperitoneally injected with control, IL-2 (50,000 IU per mouse) or anti-PD-1 (100 μg per mouse) (Supplementary information, Fig. S7d). Tumor sizes were measured every two days by caliper from day 5. WT: *March5*^*+/f*^, CHZ: *March5*^*+/f:Vav1-Cre*^. Graph shows mean ± SEM, *n* = 8. Data were analyzed using two-way ANOVA with GraphPad Prism 8. **g** Combination of IL-2 and PD-1 blockade increases the survival rate in *March5*^*+/f:Vav1-Cre*^ mice. *March5*^*+/f*^ and *March5*^*+/f:Vav1-Cre*^ mice were subcutaneously injected with MC38 cells (5 × 10^5^). On day 5 after tumor cell inoculation, mice were intraperitoneally injected with control, IL-2 (50,000 IU per mouse) or anti-PD-1 (100 μg per mouse) (Supplementary information, Fig. S7d). Mice were sacrificed when the tumor size was bigger than 15 mm of the mean tumor diameter, tumor volume exceeded 2000 mm^3^, or tumor had ulcers with diameter reached 10 mm. Statistical analysis was performed using the GraphPad Prism 8 software, *n* = 8. Kaplan–Meier survival curves and corresponding log-rank (Mantel-Cox) tests were used to evaluate the statistical differences between groups in survival studies. There is a significant difference when the *P* < 0.05.

It has been reported that *March5* homozygous deletion is embryonically lethal.^42^ To investigate the physiological functions of MARCH5, *March5*^*flox/flox*^ mice were generated and crossed with *Vav1-Cre* mice to obtain *March5* hematopoietic-specific knockout strain (*March5*^*f/f:Vav1-Cre*^). Unexpectedly, the *March5*^*f/f:Vav1-Cre*^ mice were born normally, but all died during 4–6 weeks after birth. Thus, we used *March5*^*+/f*^ and *March5*^*+/f:Vav1-Cre*^ mice for further investigation. We verified that the mRNA and protein levels of MARCH5 in the bone marrow (BM), spleen and thymus of *March5*^*+/f:Vav1-Cre*^ mice were about half to that in *March5*^*+/f*^ mice (Supplementary information, Fig. S6a, b). Flow cytometry analysis indicated that MARCH5 knockdown up-regulated the level of γ_c_ in CD4^+^ T, CD8^+^ T, NK and B cells from the spleen (Fig. 6a). MARCH5 knockdown had no marked effects on the percentages of double-negative (DN), double-positive (DP), CD4^+^ single-positive (CD4SP) cells, but increased the percentage of CD8^+^ single-positive (CD8SP) cells in the thymus (Fig. 6b; Supplementary information, Fig. S6c). *March5*^*+/f:Vav1-Cre*^ mice exhibited significantly higher percentages of CD8^+^ T and NK cells and slightly lower percentages of B and CD4^+^ T cells in spleen and the peripheral blood (Fig. 6c; Supplementary information, Fig. S6d). The percentage of CD44^high^CD62L^high^ central memory (CM) CD8^+^ T cells was also increased in total CD8^+^ T cells of spleen and the peripheral blood of *March5*^*+/f:Vav1-Cre*^ mice (Fig. 6c). The percentage of CD44^high^CD62L^high^ central memory CD4^+^ T cells did not show significant difference in CD4^+^ T cells of spleen and the peripheral blood of *March5*^*+/f:Vav1-Cre*^ mice (Supplementary information, Fig. S6d). These results suggest that MARCH5 negatively regulates γ_c_ level as well as CD8^+^ T and NK cell development in mice.

We next used mouse MC38 colorectal carcinoma and B16F10 melanoma models to investigate the biological functions of MARCH5 in anti-tumor immunity. In these models, *March5*^*+/f:Vav1-Cre*^ mice showed slower tumor progression than *March5*^*+/f*^ mice (Fig. 6d; Supplementary information, Fig. S7a). We isolated tumor-infiltrating lymphocytes (TILs) from the tumor tissues of *March5*^*+/f*^ and *March5*^*+/f:Vav1-Cre*^ mice and analyzed the immunological changes in tumors. The results showed that *March5*^*+/f:Vav1-Cre*^ mice exhibited significantly higher percentages of CD8^+^ T, NK and central memory CD8^+^ T cells in TILs comparing to *March5*^*+/f*^ mice (Fig. 6e), while the percentages of CD4^+^ T, Treg and B cells in TILs were similar between *March5*^*+/f*^ and *March5*^*+/f:Vav1-Cre*^ mice (Supplementary information, Fig. S7b, c). To determine whether MARCH5 knockdown affects the activation of tumor-infiltrating CD8^+^ T cells or the profile of exhausted T cells, we examined the T cell activation maker Granzyme B (GzmB) and the exhausted T cell marker TIM3 on infiltrated CD8^+^ T cells in the mouse tumor models. The results showed that the percentage of TIM3^+^ cells in CD8^+^ T cells from the *March5*^*+/f:Vav1-Cre*^ mice was down-regulated, while the percentage of GzmB^+^ cells in CD8^+^ T cells did not show marked changes (Supplementary information, Fig. S7b, c), suggesting that knockdown of MARCH5 inhibited exhaustion of CD8^+^ T cells and did not affect the CD8^+^ T cell response. Taken together, these data suggest that MARCH5 negatively regulates anti-tumor immunity in mouse models.

Based on their powerful abilities to stimulate proliferation of cytotoxic CD8^+^ T and NK cells, certain γ_c_ family cytokines such as IL-2 have long been used in clinical cancer immunotherapy.^27^ Since MARCH5 knockdown leads to higher level of γ_c_ in CD8^+^ T and NK cells, we reasoned that this would improve the efficacy of the γ_c_ family cytokines in anti-tumor immunotherapy. To test this, we used the B16F10 melanoma and MC38 colon cancer mouse tumor models. Administration of IL-2 in *March5*^*+/f:Vav1-Cre*^ mice showed increased efficacy in suppression of tumor growth and improvement of the overall survival comparing to that in *March5*^*+/f*^ mice (Fig. 6f, g; Supplementary information, Fig. S7d). We further combined IL-2 with PD-1 blocking antibody for tumor immunotherapy in *March5*^*+/f*^ and *March5*^*+/f: Vav1-Cre*^ mice. The results showed that the anti-tumor effects of combined IL-2 with PD-1 blockade in *March5*^*+/f:Vav1-Cre*^ mice were superior to that in *March5*^*+/f*^ mice, which eliminated tumors in 2 out of 8 *March5*^*+/f:Vav1-Cre*^ mice (Fig. 6f, g). In addition, MARCH5-deficiency in B16F10 and MC38 cells had no marked effects on their proliferation (Supplementary information, Fig. S8). Taken together, these results suggest that MARCH5 knockdown sensitizes the anti-tumor effects of IL-2 as well as its combination with PD-1 blocking antibody.

### Combination of a MARCH5 inhibitor with IL-2 and PD-1 blockade significantly increases anti-tumor efficacy

**Fig. 7:**
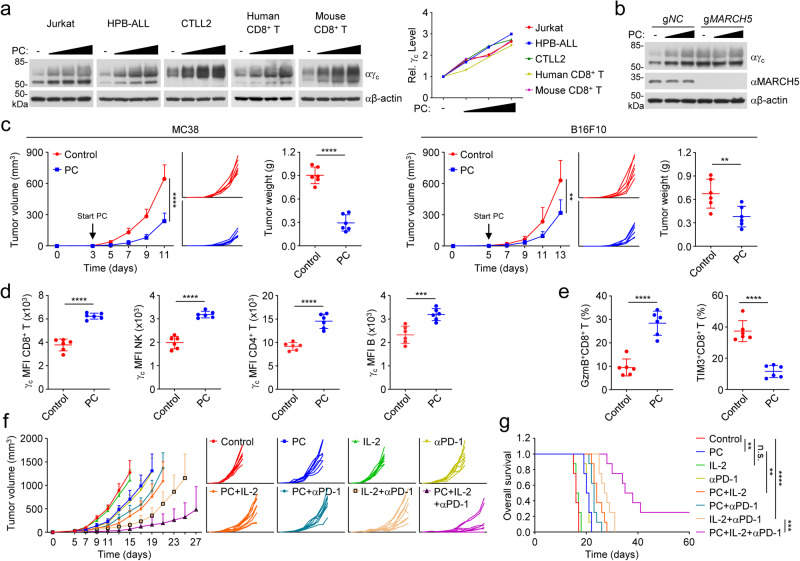
Pitavastatin calcium potentiates anti-tumor immunity triggered by combination therapy of IL-2 and PD-1 blockade. **a** PC treatment up-regulates the level of γ_c_. Human CD8^+^ T, mouse CD8^+^ T, Jurkat, HBP-ALL or CTLL2 cells were treated with PC (0, 0.5, 1, 2 μM) for 24 h before immunoblotting analysis with the indicated antibodies. The γ_c_ band intensities relative to the corresponding β-actin bands were shown in the histograph. **b** MARCH5-deficiency impairs PC-induced up-regulation of γ_c_. Control or MARCH5-deficient Jurkat cells were treated with PC (0, 0.5, 1 μM) for 24 h before immunoblotting analysis with the indicated antibodies. The immunoblots were repeated for two times with similar results. **c** PC treatment suppresses tumor growth. C57BL/6J mice were subcutaneously injected with 5 × 10^5^ of MC38 or B16F10 cells. On day 3 (MC38) or 5 (B16F10) after tumor cell implantation, mice were intraperitoneally injected with control or PC (5 mg/kg/day). Tumor sizes were measured every two days by caliper. Tumor-bearing mice were euthanized on day 13 (MC38) or day 15 (B16F10). Tumor weights were measured by Analytical Balance. Graph shows mean ± SEM, *n* = 6. Data were analyzed using Student’s unpaired *t*-test with GraphPad Prism 8. **d** Effects of PC on the level of γ_c_ in TILs. TILs were isolated from the MC38 tumor tissues in **c**. TILs were stained with the indicated antibodies and analyzed by flow cytometry. Graph shows mean ± SEM, *n* = 6 independent samples. Data were analyzed using a Student’s unpaired *t*-test with GraphPad Prism 8. MFI, median fluorescence intensities. **e** PC treatment increases tumor infiltrating CD8^+^ cytotoxic T cells. TILs were isolated from the MC38 tumor tissues in **c**. TILs were stained with the indicated antibodies and analyzed by flow cytometry. Graph shows mean ± SEM, *n* = 6 independent samples. Data were analyzed using Student’s unpaired *t*-test with GraphPad Prism 8. **f** PC potentiates the anti-tumor efficacy of IL-2 and PD-1 combination. C57BL/6J mice were subcutaneously injected with MC38 cells (5 × 10^5^). On day 5 after tumor cell implantation, mice were intraperitoneally injected with control, PC (5 mg/kg), IL-2 (50,000 IU per mouse) or anti-PD-1 (100 μg per mouse) (Supplementary information, Fig. S10b). Tumor sizes were measured every two days by caliper from day 5. Graph shows mean ± SEM, *n* = 8. Data were analyzed using two-way ANOVA with GraphPad Prism 8. **g** PC promotes the survival rate of mice treated with IL-2 and PD-1 blockade. C57BL/6J mice were subcutaneously injected with MC38 cells (5 × 10^5^). On day 5 after tumor cell implantation, mice were intraperitoneally injected with control, PC (5 mg/kg), IL-2 (50,000 IU per mouse) or anti-PD-1 (100 μg per mouse) (Supplementary information, Fig. S10b). Mice were sacrificed when the tumor size is bigger than 15 mm of the mean tumor diameter, tumor volume exceeded 2000 mm^3^, or tumor had ulcers with diameter reached 10 mm. Statistical analysis was performed using the GraphPad Prism 8 software, *n* = 8. Kaplan–Meier survival curves and corresponding log-rank (Mantel-Cox) tests were used to evaluate the statistical differences between groups in survival studies. There is a significant difference when the *P* < 0.05.

Our results reveal an important role of MARCH5 in PD-1-triggered immune suppression, which suggest that MARCH5 is a potential target for cancer immunotherapy. Unfortunately, no pharmacological inhibitors of MARCH5 have been reported. We designed a reporter system in which HEK293 cells are co-transfected with two plasmids encoding MARCH5 and γ_c_-luciferase fusion protein respectively. In this system, inhibition of MARCH5 by an inhibitor would result in increased luciferase activity in reporter assays (Supplementary information, Fig. S9a). Using this system, we screened a collection of Food and Drug Administration-approved drugs and identified 10 compounds that increased luciferase activity to more than 1.5-folds (Supplementary information, Fig. S9b and Table S2). Further confirmative experiments identified Pitavastatin calcium (PC) as the most potent inhibitor that specifically increased the luciferase activity in MARCH5-expressing but not control HEK293 cells (Supplementary information, Fig. S9c). Pitavastatin is a unique lipophilic statin and potent inhibitor of HMG-CoA reductase with a strong effect on lowering plasma total cholesterol and triacylglycerol levels. Pitavastatin has also been reported to have pleiotropic beneficial effects such as suppression of inflammation, regulation of angiogenesis and osteogenesis, improvement of endothelial function and arterial stiffness.^43^ Recent studies have also shown that PC has anti-tumor activity by promoting tumor cell apoptosis.^44,45^ Consistent with a specific inhibition of MARCH5 activity in the reporter system, PC treatment up-regulated the level of γ_c_ in a dose-dependent manner in all examined cells including primary human and mouse CD8^+^ T cells, and Jurkat, HPB-ALL and CTLL2 cells (Fig. 7a). It has been shown that PC as well as other statin drugs such as Lovastatin, Simvastatin, Fluvastatin Sodium and Rosuvastatin Calcium target HMG-CoA reductase.^46^ Therefore, we examined the effects of other statin drugs on γ_c_ level. The results indicated that only PC but not the other examined statin drugs including Lovastatin, Simvastatin, Fluvastatin Sodium and Rosuvastatin Calcium up-regulated γ_c_ level (Supplementary information, Fig. S9d), suggesting that inhibition of HMG-CoA reductase does not have an effect on γ_c_ level. In MARCH5-deficient cells, the basal level of γ_c_ was increased and PC treatment did not further increase its protein level (Fig. 7b). There results suggest that PC is a specific inhibitor of MARCH5 and capable of inhibiting MARCH5-mediated γ_c_ degradation independent of its inhibition of HMG-CoA reductase.

We further tested the anti-tumor effects of PC in mouse MC38 colorectal carcinoma and B16F10 melanoma models. Administration of PC alone significantly suppressed tumor growth in mice (Fig. 7c). Administration of PC also resulted in higher level of γ_c_ in tumor-infiltrating lymphocytes (Fig. 7d). The percentages of CD8^+^ T, NK, CD4^+^ T, Treg and B cells in tumor-infiltrating lymphocytes did not show significant difference after PC administration (Supplementary information, Fig. S10a). PC treatment up-regulated the percentage of GzmB^+^ cells and reduced the percentage of TIM3^+^ cells in infiltrated CD8^+^ T cells, suggesting that PC enhances infiltration of CD8^+^ cytotoxic T cells and inhibits exhaustion of CD8^+^ T cells (Fig. 7e).

We further investigated the anti-tumor effects of PC combined with IL-2 and PD-1 blockade (Supplementary information, Fig. S10b). Consistently, PC significantly increased the efficacies of IL-2 or IL-2 plus PD-1 blocking antibody on tumor suppression (Fig. 7f) and overall survival of mice (Fig. 7g). In addition, we investigated whether the other HMG-CoA reductase inhibitors such as Rosuvastatin Calcium affect the anti-tumor efficacies of IL-2. Administration of PC or Rosuvastatin Calcium alone suppressed tumor growth in mice. However, only PC but not Rosuvastatin Calcium has a synergistic effect with IL-2 on tumor suppression (Supplementary information, Fig. S10c), suggesting that PC promotes anti-tumor immunity via its inhibition of MARCH5 but not HMG-CoA reductase. Taken together, these results suggest that administration of MARCH5 inhibitor leads to increased efficacies of cancer immunotherapy by IL-2 plus PD-1 blockade.

## Discussion

Combination therapy with PD-1 blockade and IL-2 substantially improves anti-tumor efficacy compared with PD-1 blockade or IL-2 monotherapy, which has been extensively explored for immunotherapy of various cancers.^15,21,26,47^ The basic principle of combined immunotherapy with PD-1 blockade and IL-2 is to remove the PD-1 inhibitory brake and in the meantime provide a stimulatory signal for cytotoxic T lymphocytes with IL-2. Recent studies have also shown that combination immunotherapy with PD-1 blockade and IL-2 modifies CD8^+^ T cell exhaustion program.^47,48^ Currently, the cellular and molecular basis responsible for the synergistic effects observed between PD-1 blockade and IL-2 remains enigmatic. In this study, we showed that PD-1 signaling negatively regulates the stability and activity of γ_c_, which impairs the γ_c_ family cytokine-triggered signaling and immune activation of CD8^+^ T cells in the TME. PD-1 blockade removes the inhibitory effects on γ_c_, thereby restoring the responses of CD8^+^ T cells to the γ_c_ family cytokines such as IL-2 and leading to the synergistic effects of combination immunotherapy of PD-1 blockade and IL-2. Most importantly, we uncovered the two distinct molecular mechanisms responsible for PD-1 signaling-triggered inhibition of γ_c_-mediated immune activation, and validated the membrane-associated E3 ligase MARCH5 as a potential target for combination immunotherapy.

PD-1 is a key coinhibitory receptor that inhibits the killing function of cytotoxic lymphocytes upon engagement with its ligands (PD-L1 or PD-L2).^49–53^ To evade host immune surveillance, tumor cells take advantage of this inhibitory pathway by overexpressing PD-L1.^12,54^ γ_c_ is a component of the receptors for IL-2, IL-4, IL-7, IL-9, IL-15 and IL-21, which is constitutively expressed on various populations of immune cells.^19^ In our experiments, we found that the level of γ_c_ in TME was negatively correlated with PD-L1. PD-1 blockade in mouse tumor model up-regulated the level of γ_c_ in tumor-infiltrating CD8^+^ T cells. It has been shown that expression of γ_c_ family cytokine receptors, such as IL2Rα and IL2Rβ, is induced by extracellular stimuli such as antigens, mitogens and cytokines, whereas γ_c_ is not induced.^19,55–57^ Our results confirmed that the level of γ_c_ was not markedly changed after T cell activation or γ_c_ family cytokine stimulation. However, PD-1 ligation promoted lysosomal degradation of γ_c_.

In a screen for potential E3 ubiquitin ligases that mediate γ_c_ degradation, we identified MARCH5, which is a member of the membrane-associated E3 ubiquitin ligase family.^35^ Previous studies have demonstrated that MARCH5 is involved in regulation of mitochondrial morphology and pexophagy.^36,37,58^ Our results indicated that MARCH5 was also colocalized with γ_c_ at the plasma membrane and intracellular membrane organelles such as endosome and lysosome. Endogenous coimmunoprecipitation experiments indicated that MARCH5 was associated with γ_c_, which was increased following PD-L1 stimulation. Overexpression of MARCH5 but not its inactive mutant MARCH5^H43W^ promoted K27-linked polyubiquitination and lysosomal degradation of γ_c_. MARCH5-deficiency up-regulated the level of γ_c_, which was reversed by reconstitution with wild-type MARCH5 but not MARCH5^H43W^. PD-1 ligation induced K27-linked polyubiquitination and lysosomal degradation of γ_c_, which were blocked in MARCH5-deficient cells. In contrast, PD-1 ligation had no marked effects on the protein level of γ_c_ in MARCH5-deficient cells reconstituted with ectopically-expressed wild-type MARCH5. These results suggest that PD-1 signaling leads to MARCH5-mediated K27-linked polyubiquitination and degradation of γ_c_, but does not affect MARCH5 activity per se. Mechanistically, we found that PD-1 ligation induced the transcription factor BATF, which transcriptionally induced MARCH5 by binding to the promoter of *MARCH5* gene. BATF-deficiency impaired PD-L1-induced up-regulation of *MARCH5* mRNA and down-regulation of γ_c_ protein level. Our experiments suggest that PD-1 signaling induces the transcription factor BATF, which in turn induces expression of the E3 ubiquitin ligase MARCH5, leading to K27-linked polyubiquitination and lysosomal degradation of γ_c_.

Our experiments further identified USP5 as an enzyme that constitutively deubiquitinates γ_c_. USP5 removed K27-linked polyubiquitin moieties of γ_c_ conjugated by MARCH5. Knockout of USP5 increased PD-1 ligation-induced K27-linked polyubiquitination of γ_c_ and down-regulated its protein level in cells. These results suggest that USP5 acts as a constitutive guard for γ_c_ stability to ensure proper responses of CD8^+^ T cells to the γ_c_ family cytokines. Consistently, our experiments showed that MARCH5-deficiency potentiated the γ_c_ family cytokine-triggered signaling and immune activation, whereas USP5-deficiency had the opposite effects. Taken together, our experiments suggest that the BATF-MARCH5-γ_c_ axis mediates PD-1-triggered inhibition of γ_c_ family cytokine-triggered signaling and immune activation.

Our experiments also suggest that SHP2 mediates another mechanism responsible for PD-1-triggered inhibition of γ_c_ family cytokine-triggered signaling and immune activation. Previously, it has been shown that PD-1 ligation triggers clustering of PD-1 with TCR, and PD-1 is phosphorylated by TCR proximal Src family kinases.^13,31,33^ Phosphorylation of PD-1 recruits and activates the allosteric dephosphorylating enzyme SHP2, which mediates dephosphorylation of TCR proximal signaling molecules. The clustering of PD-1 and TCR appears to be required for PD-1 phosphorylation and SHP2 recruitment. However, PD-1 is partially segregated from TCR after clustering, while it interacts with SHP2 constitutively,^30,33,59^ suggesting that PD-1-associated SHP2 has the potential to mediate dephosphorylation of molecules that is not part of the TCR signalosome. Consistently, T cell costimulatory receptor CD28, which is not a TCR-associated component, is dephosphorylated by PD-1-SHP2.^30^ In our experiments, we found that PD-1 ligation promoted the association of γ_c_ with SHP2. Overexpression of gain-of-function SHP2 mutants (D61G or E76K) reduced JAK3-mediated phosphorylation of γ_c_^Y357^. Knockout of SHP2 impaired PD-1 ligation-induced γ_c_^Y357^ dephosphorylation. Knockout of SHP2 also increased the γ_c_ family cytokine-induced phosphorylation of γ_c_^Y357^ and STAT5^Y694/Y699^. Reconstitution of wild-type γ_c_ but not γ_c_^Y357F^ in γ_c_-deficient HPB-ALL cells restored IL-7- and IL-9-induced phosphorylation of STAT5^Y694/Y699^. These results suggest that PD-1 ligation-triggered SHP2 activation induces dephosphorylation of γ_c_^Y357^, resulting in desensitization of γ_c_-mediated signaling and immune activation. In our study, we found that the protein levels of γ_c_ in MARCH5-deficient cells reconstituted with ectopically-expressed wild-type MARCH5 did not markedly changed after PD-1 ligation, suggesting that SHP2-mediated dephosphorylation of γ_c_ is not involved in MARCH5-mediated down-regulation of γ_c_.

**Fig. 8:**
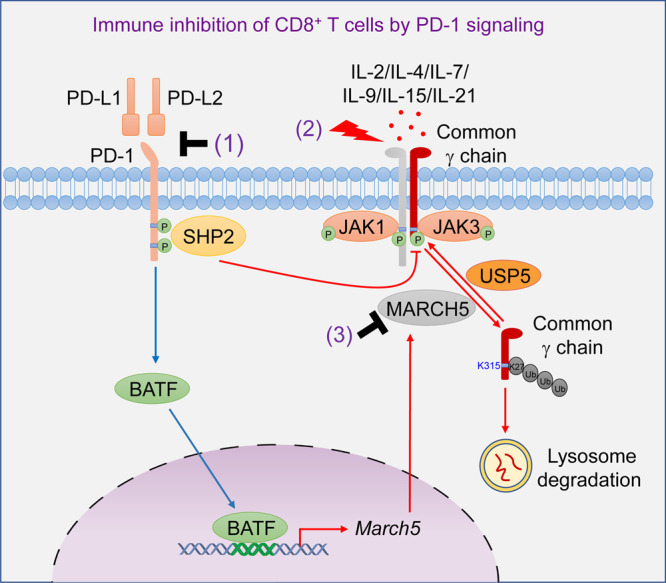
A model on regulation of γ_c_ stability and activity by PD-1 signaling. In tumor microenvironment, PD-L1/PD-1 signaling results in inhibition of the γ_c_ family cytokine-triggered signaling and immune activation by two mechanisms. Immediately after PD-1 ligation, SHP2 is recruited to PD-1 and activated, which in turn dephosphorylates γ_c_ at Y357, leading to its inactivation and unresponsiveness to γ_c_ family cytokines. More later after PD-1 ligation, the transcription factor BATF is induced, which up-regulates the expression of the membrane-associated E3 ubiquitin ligase MARCH5. MARCH5 is recruited to γ_c_ and mediates its K27-linked polyubiquitination at K315 and lysosomal degradation. Targeting of components involved in these regulatory mechanisms, such as by a combination of PD-1 blocking antibody (1), IL-2 (2) and MARCH5 inhibitor (3) leads to potent anti-tumor immunity.

Based on our results, we propose a model on the regulatory mechanisms of γ_c_ stability and activity by PD-1 signaling. In TME with high expression of PD-L1 in tumor cells, PD-1 signaling in immune cells is hijacked and activated. The activated PD-1 recruits and activates SHP2, which subsequently mediates dephosphorylation of γ_c_^Y357^, leading to its inactivation and unresponsiveness to γ_c_ family cytokines. On the other hand, PD-1 signaling induces the transcription factor BATF, which induces expression of the membrane-associated E3 ubiquitin ligase MARCH5. MARCH5 is recruited to γ_c_ and mediates its K27-linked polyubiquitination at K315 and lysosomal degradation. Therefore, PD-1 signaling suppresses the γ_c_ family cytokine-triggered immune activation via two distinct mechanisms. As shown in our experiments, PD-1-triggered SHP2 activation and dephosphorylation of γ_c_ occurred in minutes, whereas PD-1-triggered induction of MARCH5 and degradation of γ_c_ was obvious one day after PD-1 ligation; thus, we propose that PD-1 signaling inhibits γ_c_ family cytokine-triggered immune activation via the two mechanisms in a temporal manner. In this context, it has previously been shown that PD-1 signaling causes dephosphorylation of TCR proximal molecules and subsequent transcriptional inhibition.^13,41,59^ Targeting of components involved in these regulatory mechanisms, such as SHP2, MARCH5 and USP5, may increase the efficacy of cancer immunotherapy by PD-1 blockade and the γ_c_ family cytokines (Fig. 8). Consistently, our experiments indicated that MARCH5-knockdown up-regulated the level of γ_c_ in lymphocytes, increased the percentage of CD8^+^ single-positive (CD8SP) cells in thymocytes and enhanced the percentages of CD8^+^ T and NK cells in the spleen and peripheral blood in mice. MARCH5-knockdown suppressed tumor growth, and sensitized the anti-tumor effects of IL-2 as well as its combination with PD-1 blocking antibody in mice.

In our study, we also identified PC as an inhibitor of MARCH5. Our experiments indicated that PC up-regulated the level of γ_c_ in a MARCH5-dependent manner. In MARCH5-deficient cells, the basal level of γ_c_ was increased and PC treatment did not further increase its protein level. These results suggest that PC inhibits MARCH5 activity. PC is a unique lipophilic statin and potent inhibitor of HMG-CoA reductase. PC but not the other HMG-CoA reductase inhibitors such as Lovastatin, Simvastatin, Fluvastatin Sodium and Rosuvastatin Calcium treatment up-regulated the level of γ_c_, suggesting that inhibition of HMG-CoA reductase does not have an effect on γ_c_ level and PC-induced down-regulation of γ_c_ is independent of its inhibition of HMG-CoA reductase. Consistently, PC treatment up-regulated the level of γ_c_ in tumor-infiltrating lymphocytes, inhibited tumor growth, and sensitized the anti-tumor effects of IL-2 as well as its combination with PD-1 blocking antibody. In addition, only PC but not Rosuvastatin Calcium had a synergistic effect with IL-2 on tumor suppression, suggesting that PC promotes anti-tumor immunity via its inhibition of MARCH5 but not HMG-CoA reductase. Previous studies have demonstrated that MARCH5 inhibits mitochondrial fission by promoting Drp1 degradation,^36,60^ whereas other studies suggest that MARCH5 is required for Drp1-dependent mitochondrial division.^58,61^ In addition, it has also been reported that MARCH5 is partially localized at the peroxisome to regulate pexophagy.^37^ Whether and how the functions of MARCH5 in these cellular processes other than its down-regulation of γ_c_ are involved in its regulation of anti-tumor immunity needs to be investigated in future studies. Given its important role of in regulating γ_c_ stability, USP5 can be a potential target for cancer immunotherapy. Unfortunately, USP5 is aberrantly expressed in human cancers and promotes tumor growth and metastasis,^62–64^ which may limit its application as a target for cancer immunotherapy.

In conclusion, our current study suggests that MARCH5-mediated degradation and SHP2-mediated inactivation of γ_c_ triggered by PD-1 signaling render CD8^+^ T cells in TME irresponsive to immune activation by the γ_c_ family of cytokines. PD-1 blockade therapy restores γ_c_ stability and activity, thereby sensitizing CD8^+^ T cells to IL-2. Our findings define the underlying mechanisms on how IL-2 combined with PD-1 blockade can be synergistic in tumor immunotherapy. As validated in our study, regulation of these mechanisms would provide new strategies for improved efficacy of combination immunotherapy of cancer.

## Materials and methods

### Reagents and antibodies

Reagents and antibodies used in this study were purchased from the indicated companies: recombinant human PD-L1-Fc fusion protein (BPS Bioscience, Catalog #71104), recombinant mouse PD-L1-Fc fusion protein (Sino, Catalog #50010-M02H), anti-human CD3ε (clone OKT3, Biolegend, Catalog #317326), anti-human CD28 (clone CD28.2, Biolegend, Catalog #302934), anti-mouse CD3ε (clone 145-2C11, Biolegend, Catalog #100340), anti-mouse CD28 (clone 37.51, Biolegend, Catalog #102116), PHA (Sigma, Catalog #L8954), polybrene (Millipore, Catalog #3924803), SYBR (Bio-Rad, Catalog #1725125), cycloheximide (Sigma, Catalog #239763), MG132 (Sigma, Catalog #M8699), NH_4_Cl (Sigma, Catalog #254134), 3-MA (Sigma, Catalog #189490), Pitavastatin calcium (Aladdin, Catalog #P129617), Rosuvastatin calcium (Aladdin, Catalog #R129220), Simvastatin (Aladdin, Catalog #S129538), Lovastatin (Aladdin, Catalog #L107709), Fluvastatin Sodium (Aladdin, Catalog #F129852), human IL-2 (SL Pharm, Catalog #S19991010), human IL-7 (Peprotech, Catalog #200-07), mouse IL-7 (Peprotech, Catalog #217-17) and human IL-9 (Peprotech, Catalog #200-09). Information on the commercially available antibodies used in this study is provided in Supplementary information, Table S3. The antibody that specifically recognizes phosphorylated Y357 of γ_c_ was raised by immunizing rabbits with a synthetic peptide of human γ_c_ (_354_HSP(Y-p)WAPPC_362_) by ABclonal Technology (Wuhan).

### Cells

Jurkat cells were obtained from American Type Culture Collection. HEK293 cells were originally provided by Dr. Gary Johnson (National Jewish Health, Denver, CO). HPB-ALL cells were provided by Dr. Hudan Liu (Wuhan university). CTLL2 cells were obtained from Cell Resource Center (IBMS, CAMS/PUMC). Human CD8^+^ T cells were obtained from Milestone® Biotechnologies. Mouse CD8^+^ T cells were isolated from the spleen of 6–8-week-old C57BL/6 mice by negative selection magnetic beads (STEM CELL Technologies, Catalog #19853 A). B16F10 cells, CT26 cells and MC38 cells were provided by Dr. Jinfang Zhang (Wuhan University).

Jurkat cells were cultured in RPMI 1640 (GIBCO, Catalog #C11875500BT) supplemented with 10% FBS (Cell Max, Catalog #SA211.02) and 1% penicillin-streptomycin (GIBCO, Catalog #15140-122). HPB-ALL cells were cultured in RPMI 1640 supplemented with 10% FBS, 1% penicillin-streptomycin, 1% non-essential amino acids (GIBCO, Catalog #11140-050), 2 mM _L_-glutamine (GIBCO, Catalog #35050-061), 1 mM sodium pyruvate (GIBCO, Catalog #11360-070) and 55 μM β-mercaptoethanol (Sigma-Aldrich, Catalog #63689). CTLL2 cells were cultured in RPMI 1640 supplemented with 10% FBS, 1% penicillin-streptomycin and IL-2 (100 IU/mL). Human CD8^+^ T cells and mouse CD8^+^ T cells were cultured in RPMI 1640 supplemented with 10% FBS, 1% penicillin-streptomycin, 1% non-essential amino acids, 2 mM _L_-glutamine, 1 mM sodium pyruvate, 55 μM β-mercaptoethanol and IL-2 (400 IU/mL). HEK293, B16F10, CT26 and MC38 cells were cultured in DMEM (GIBCO) supplemented with 10% FBS and 1% penicillin-streptomycin. All cells were detected negative for mycoplasma.

### Constructs

Mammalian expression plasmids for Flag-, HA-, or Myc-tagged γ_c_, MARCH5, β-actin, IL2RB, IL4R, IL7R, OTUD6B, OTULIN, UCHL5, USP3, USP5, USP11, USP14, SHP2, JAK3 and their mutants, as well as pSuper.Retro-shRNA plasmids for MARCH5 were constructed by standard molecular biology techniques. Guide-RNA plasmids targeting γ_c_, MARCH5, USP5, BATF and SHP2 were constructed into a lentiCRISPR V2 vector, which was provided by Dr. Shu-Wen Wu (Wuhan University).

### Transfection

HEK293 cells were transfected by standard calcium phosphate precipitation. The empty control plasmid was added to ensure that each transfection receives the same amount of total DNA.

### CRISPR-Cas9 knockout

Double-stranded oligonucleotides corresponding to the target sequences were cloned into the Lenti-CRISPR-V2 vector, which were co-transfected with the packaging plasmids into HEK293 cells. Two days after transfection, the viruses were harvested, ultra-filtrated (0.45-μm filter, Millipore) and used to infect cells in the presence of polybrene (8 μg/mL). The infected cells were selected with puromycin (Jurkat: 1 μg/mL, HPB-ALL: 2 μg/mL, CTLL2: 4 μg/mL) for at least 6 days. The information of gRNA sequences is shown in Supplementary information, Table S4.

### Co-immunoprecipitation, ubiquitination and immunoblotting analysis

Cells were lysed in 1 mL of NP-40 lysis buffer (20 mM Tris-HCl, 150 mM NaCl, 1 mM EDTA, 1% Nonidet P-40, 1% Triton X-100, 10 μg/mL aprotinin, 10 μg/mL leupeptin, and 1 mM phenylmethylsulfonyl fluoride, PMSF). For each immunoprecipitation reaction, a 0.4 mL aliquot of lysate was incubated with 0.5–2 μg of the indicated antibody or control IgG and 35 μL of a 1:1 slurry of Protein-G Sepharose at 4 °C for 3 h. The Sepharose beads were washed three times with 1 mL of lysis buffer containing 500 mM NaCl. The precipitates were fractionated by SDS-PAGE, and immunoblotting analysis was performed with the indicated antibodies. For ubiquitination assays, the immunoprecipitants were re-extracted in NP-40 lysis buffer containing 1% SDS and denatured by heating for 10 min. The supernatants were diluted with regular lysis buffer until the concentration of SDS was decreased to 0.1%, following by re-immunoprecipitation with the indicated antibodies. The immunoprecipitants were analyzed by immunoblotting with the ubiquitin antibody.

### qPCR

Total RNA was isolated for qPCR analysis to measure mRNA abundance of the indicated genes. Data shown are the relative abundance of the indicated mRNAs normalized to that of GAPDH. The qPCR data were collected with Bio-Rad CFX96 (Version 3.1) and analyzed with Bio-Rad CFX Manager (Version 3.1). Gene-specific primer sequences are listed in Supplementary information, Table S5.

### ChIP

ChIP was performed according to the manufacture’s instruction. Ten million cells were fixed with 1% formaldehyde for 10 min, quenched with 0.125 M glycine for 5 min at 37 °C and lysed in SDS Lysis Buffer. Cell lysate was sonicated by Bioruptor Pico Sonifier to shear chromatin DNA to a size range of 200–1000 bp. The supernatant was diluted 10-fold in ChIP Dilution Buffer and precleared with 60 mL agarose beads for 30 min. The supernatant fraction was immunoprecipitated with the indicated antibodies (2 μg) against BATF overnight at 4 °C. The antibody-chromatin complexes were pulled down with protein A agarose/salmon sperm DNA beads (Sigma, Catalog #16-157) for 1 h at 4 °C. The de-crosslinked DNA was subjected to qPCR analysis using specific primers listed in Supplementary information, Table S6.

### Flow cytometry analysis

Cells were subjected to stain with the indicated antibodies for 30 min in 4 °C. The cells were analyzed and data were acquired with BD Fortessa X-20 and FACSDiva 7 software following the exemplified gating strategy for flow cytometry analysis. The data were processed using FlowJo software. The antibodies used for flow cytometric analyses in this study are provided in Supplementary information, Table S7.

### Mass spectrometry

Jurkat cells (1 × 10^8^) were used for mass spectrometry analysis. Endogenous γ_c_ was immunoprecipitated with anti-γ_c_ and desalted, then analyzed by mass spectrometry. Mass spectrometry analysis was performed as previously described by SpecAlly (Wuhan) Life Science and Technology Company.^65,66^

### Proliferation assay

Cells (5 × 10^4^) were seeded in 6-cm dish for 24 h. Triplicate wells were seeded for each experimental group. The cells were trypsinized, resuspended in DMEM containing 10% FBS, and counted with a Cellometer (Bio Red) every 2 days over a 5 days period.

### Human NSCLC samples

The human NSCLC samples were provided by Dr. Bo Zhong (Wuhan University).^67^ All cases were re-reviewed by pathologists from the Department of Pathology of Tongji Hospital for the confirmation of tumor histology and tumor content. All cases used in this study were performed with written patient informed consents and approved by the Institutional Review Committee of Tongji Hospital, Tongji Medical College, Huazhong University of Science and Technology, and the Medical Ethic Committee of the School of Medicine, Wuhan University.

### IHC staining

IHC staining was performed as previously described.^68^ In brief, the slides were deparaffinized in xylene, and rehydrated sequentially in 100%, 95%, and 75% ethanol for 5 min. The antigen retrieval was performed by heating slides in a microwave for 30 min in sodium citrate buffer (pH 6.0) or 0.5 mM EDTA buffer (pH 8.0). The sections were cooled down naturally to room temperature and quenched in 3% hydrogen peroxide to block endogenous peroxidase activity. The sections were incubated with antibodies overnight at 4 °C. Next, a secondary biotinylated immunoglobulin G antibody solution and an avidin-biotin peroxidase reagent were added onto slides. After washing with phosphate buffer saline, 3,3′-diaminobenzidine tetrachloride was added to the sections, followed by counterstaining with hematoxylin (Beyotime Biotech). The information and dilutions of antibodies are listed in Supplementary information, Table S3. Signals were imaged with an Aperio VERSA 8 (Leica) multifunctional scanner and quantified with the software Image-Pro Plus 6.0.

### MARCH5 conditional knockout mice and genotyping

*March5*^*flox/flox*^ mice were generated by the Animal Center of Wuhan University Medical Research Institute. Genotyping by PCR was performed using the following primers: F, 5′-AAGGACCTCTTGAACTTGGAAAG-3′; R, 5′-GCCCATACAGTCATGTAGGCAAA-3′. Amplification of the wild-type (WT) allele with primers F and R generates a 1020-bp fragment, whereas amplification of the disrupted allele with primers F and R generates a 1088-bp fragment. To generate MARCH5 hematopoietic-specific knockout mice, *March5*^*flox/flox*^ mice were bred to *Vav1-Cre* mice to generate *March5*^*f/f:Vav1-Cre*^ mice. Genotyping of the *Vav1-Cre* mice by PCR was performed using the following primers which produces a 205-bp fragment: Forward-CGTATAGCCGAAATTGCCAG; Reverse-CAAAACAGGTAGTTATTCGG. To generate MARCH5 T cell-specific knockout mice, *March5*^*flox/flox*^ mice were bred to *CD4-Cre* mice to generate *March5*^*f/f:CD4-Cre*^ mice. Genotyping of the *CD4-Cre* mice by PCR was performed using the following primers which produces a 408-bp fragment: Forward-GCATTACCGGTCGATGCAACGAGTGATGAG; Reverse-GAGTGAACGAACCTGGTCGAAATCAGTGCG. All animal utility was carried out in compliance with the Institutional Animal Care and Use Committee (IACUC) guidelines and approved by the Animal Care and Ethics Committee of Wuhan University Medical Research Institute.

### In vivo experimental therapy in syngeneic mouse tumor models

Age- and sex-matched *March5*^*+/f*^ and *March5*^*+/f:Vav1-Cre*^ mice, C57BL/6J or Balb/c mice (all age of 6–8 weeks) were anaesthetized and subcutaneously injected with the indicated mouse tumor cells (5 × 10^5^ in 200 μL PBS). The mice were euthanized when the tumor size was bigger than 15 mm of the mean tumor diameter or tumor volume reaches 2000 mm^3^ or deemed as died.

Anti-PD-1 therapy in C57BL/6J and Balb/c mice: mice were intraperitoneally injected with control or anti-PD-1 (BE0273, 100 μg per mouse, dissolved in PBS, BioXCell) every three days (four times in total) five days after B16F10 (C57BL/6J mice) or CT26 (Balb/c mice) cells inoculation. Tumor-bearing mice were euthanized on day 17. Tumor tissues were analyzed by IHC staining and TILs were analyzed by flow cytometry.

The MC38 and B16F10 tumor models in *March5*^*+/f*^ and *March5*^*+/f:Vav1-Cre*^ mice: on the day of three after tumor cells implantation, tumor sizes were measured every two days by caliper. Tumor-bearing mice were euthanized on day 13. TILs were analyzed by flow cytometry.

PC therapy in C57BL/6J mice: mice were intraperitoneally injected with control or PC (P129617, 5 mg/kg/day, dissolved in PBS, Aladdin) three (MC38 tumor) or five (B16F10 tumor) days after inoculation of tumor cells, and tumor sizes were measured every two days by caliper. Tumor-bearing mice were euthanized on day 13 (MC38 tumor) or day 15 (B16F10 tumor). TILs were analyzed by flow cytometry.

IL-2 and anti-PD-1 therapy in *March5*^*+/f*^ and *March5*^*+/f:Vav1-Cre*^ mice: mice were intraperitoneally injected with control, IL-2 (50,000 IU per mouse, dissolved in PBS) or anti-PD-1 (100 μg per mouse, dissolved in PBS) five days after inoculation of MC38 cells. Tumor size and mouse survival were measured every two days from day 5.

PC, IL-2 and anti-PD-1 therapy in C57BL/6J mice: mice were intraperitoneally injected with control, PC (5 mg/kg, dissolved in PBS), IL-2 (50,000 IU per mouse, dissolved in PBS) and anti-PD-1 (100 μg per mouse, dissolved in PBS) five days after inoculation of MC38 cells. Tumor size and mouse survival were measured every two days from day 5.

PC, RC and IL-2 therapy in C57BL/6J mice: mice were intraperitoneally injected with PBS, PC (5 mg/kg, dissolved in PBS), RC (20 mg/kg, dissolved in PBS) and IL-2 (50,000 IU per mouse, dissolved in PBS) five days after inoculation of MC38 cells. Tumor size and mouse survival were measured every two days from day 5.

For survival studies, mice were sacrificed when the tumor size was bigger than 15 mm of the mean tumor diameter, tumor volume exceeded 2000 mm^3^, or tumor had ulcers with a diameter reached 10 mm. Statistical analysis was performed using the GraphPad Prism 8 software. Kaplan–Meier survival curves and corresponding log-rank (Mantel-Cox) tests were used to evaluate the statistical differences between groups in survival studies. There is a significant difference when the *P* < 0.05.

### Isolation of tumor-infiltrated immune cells

Tumor tissues were separated from mice and cut into pieces. The tumor tissues were suspended with 2 mL of tumor digestion buffer (1× HBSS buffer with 5 mg/mL collagenase II and 0.1% DNase I) and rotated for at 37 °C for 1 h. The cell suspension was filtered using a 70-μm filter to obtain single-cell suspension. The lymphocytes were isolated by density-gradient centrifugation using 40% and 70% Percoll (GE). The TILs were stained using fluorescently labelled antibodies for different markers. Cells were analyzed and data were acquired with BD Fortessa X-20 and FACSDiva 7 software following the exemplified gating strategy for flow cytometry analysis. The data were processed using FlowJo software.

### Confocal microscopy

H1299 or A549 cells were transfected with the indicated plasmids for 20 h. The cells were fixed with 4% paraformaldehyde for 15 min, and permeabilized with 0.3% Triton X-100 in PBS for 15 min. The cells were blocked with 5% BSA in PBS and stained with the indicated primary and secondary antibodies. The nuclei were strained with DAPI for 2 min and then washed with PBS for 3 times. The stained cells were observed with a Zeiss LSM880 confocal microscope under a 63× oil objective.

### Statistical analysis

Data were analyzed using Student’s unpaired *t*-test, multiple *t*-test or two-way ANOVA with GraphPad Prism 8. The correlation study was analyzed using a Spearman rank correlation test. The number of asterisks represents the degree of significance with respect to *P* values, with the latter presented within each figure or figure legend. All the biochemical experiments, particularly immunoblotting analysis, were repeated for at least two times with similar results. The reproducibility of other experiments is described in the respective figure legends.

### Supplementary information

Supplementary information, Fig. S1

Supplementary information, Fig. S2

Supplementary information, Fig. S3

Supplementary information, Fig. S4

Supplementary information, Fig. S5

Supplementary information, Fig. S6

Supplementary information, Fig. S7

Supplementary information, Fig. S8

Supplementary information, Fig. S9

Supplementary information, Fig. S10

Supplementary information, Table S1

Supplementary information, Table S2

Supplementary information, Table S3

Supplementary information, Table S4

Supplementary information, Table S5

Supplementary information, Table S6

Supplementary information, Table S7

## Data Availability

All the data supporting the findings of this study are available within the article and supplementary information files, or can be obtained from the corresponding author upon reasonable request.
