# Supplementary material for: PD-1 signaling negatively regulates the common cytokine receptor γ chain via MARCH5-mediated ubiquitination and degradation to suppress anti-tumor immunity
Source: Cell Res. 2023 Nov 6;33(12):923–39. doi: 10.1038/s41422-023-00890-4 (PMC10709454; doi:10.1038/s41422-023-00890-4)
Supplement: Supplementary file 1 — Supplementary information, Fig. S1 [file 41422_2023_890_MOESM1_ESM.pdf]

Supplementary information, Fig. S1. Related to Fig. 2

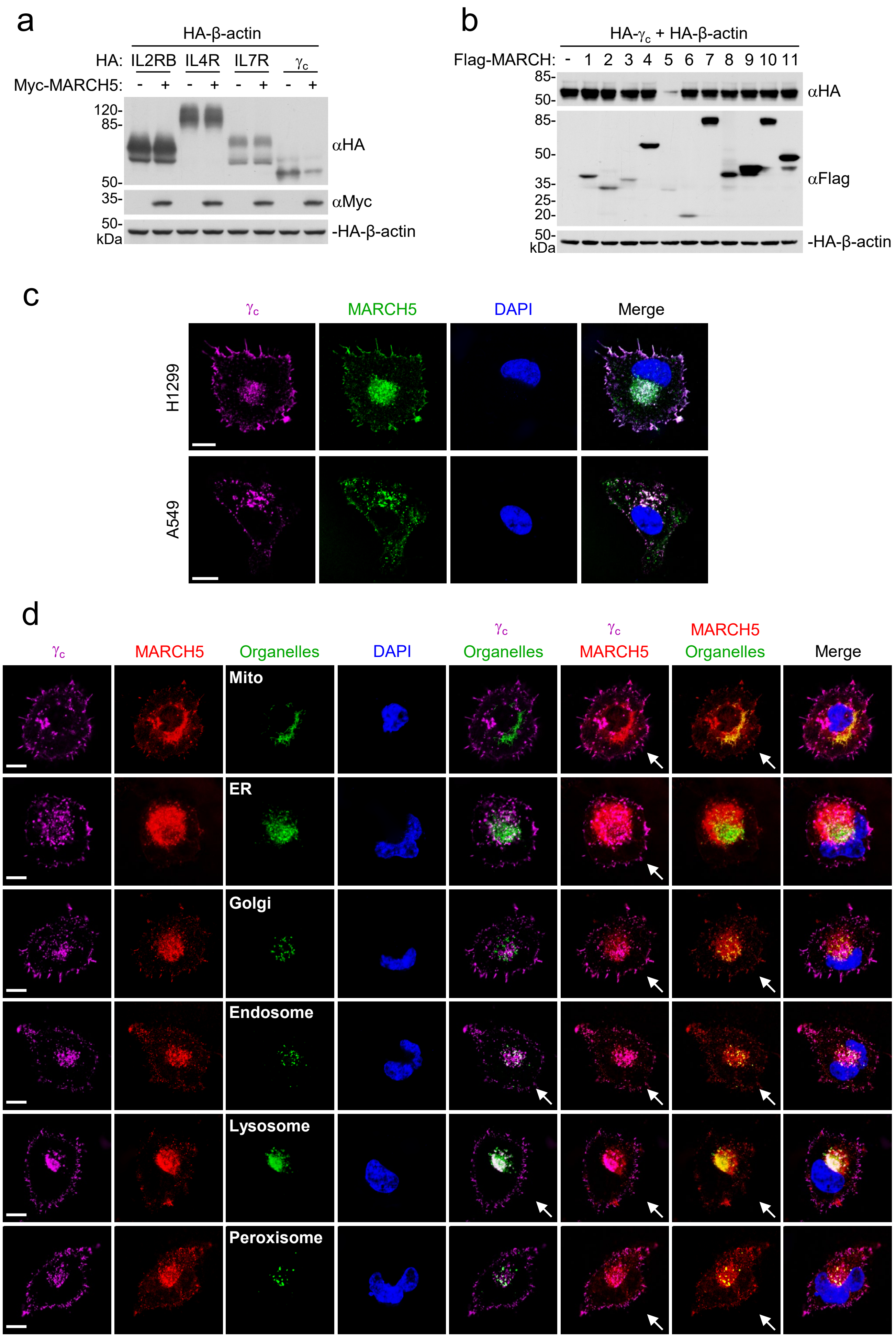

Supplementary information, Fig. S1 MARCH5 mediates K27-linked polyubiquitination and degradation of γ_c. Related to Fig. 2.

(a) MARCH5 down-regulates the level of γ_c but not the γ_c family cytokine receptors. HEK293 cells were transfected with the indicated plasmids for 24 h before immunoblotting analysis with the indicated antibodies.

(b) Effects of overexpression of MARCH proteins on γ_c protein level. HEK293 cells were transfected with the indicated plasmids for 24 h before immunoblotting analysis with the indicated antibodies.

(c) Colocalization of γ_c with MARCH5. H1299 or A549 cells were transfected with FLAG-tagged γ_c and HA-tagged MARCH5 for 20 h. The cells were fixed with 4% paraformaldehyde and stained with anti-FLAG (violet), anti-HA (green) and DAPI (blue). Images were obtained under a 63x oil objective of a confocal microscope. Representative images from IF staining are shown. Scale bar, 10 µm.

(d) Localization of γ_c and MARCH5 in the plasma membrane and organelles. H1299 cells were transfected with FLAG-tagged γ_c, MARCH5-mCherry and indicated TFP-tagged organelle marker (Mitochondria: Mito-YFP; ER: Rab7-YFP; Golgi: GalT-YFP; endosome: EEA1-YFP, lysosome: Lamp1-YFP; peroxisome: PXMP2-YFP) for 20 h. The cells were fixed with 4% paraformaldehyde and stained with anti-FLAG (violet) and DAPI (blue). Images were obtained under a 63x oil objective of a confocal microscope. Representative images from IF staining are shown. Scale bar, 10 µm.

All the experiments were repeated for at least two times with similar results
