# Supplementary material for: PD-1 signaling negatively regulates the common cytokine receptor γ chain via MARCH5-mediated ubiquitination and degradation to suppress anti-tumor immunity
Source: Cell Res. 2023 Nov 6;33(12):923–39. doi: 10.1038/s41422-023-00890-4 (PMC10709454; doi:10.1038/s41422-023-00890-4)
Supplement: Supplementary file 2 — Supplementary information, Fig. S2 [file 41422_2023_890_MOESM2_ESM.pdf]

Supplementary information, Fig. S2. Related to Fig. 2

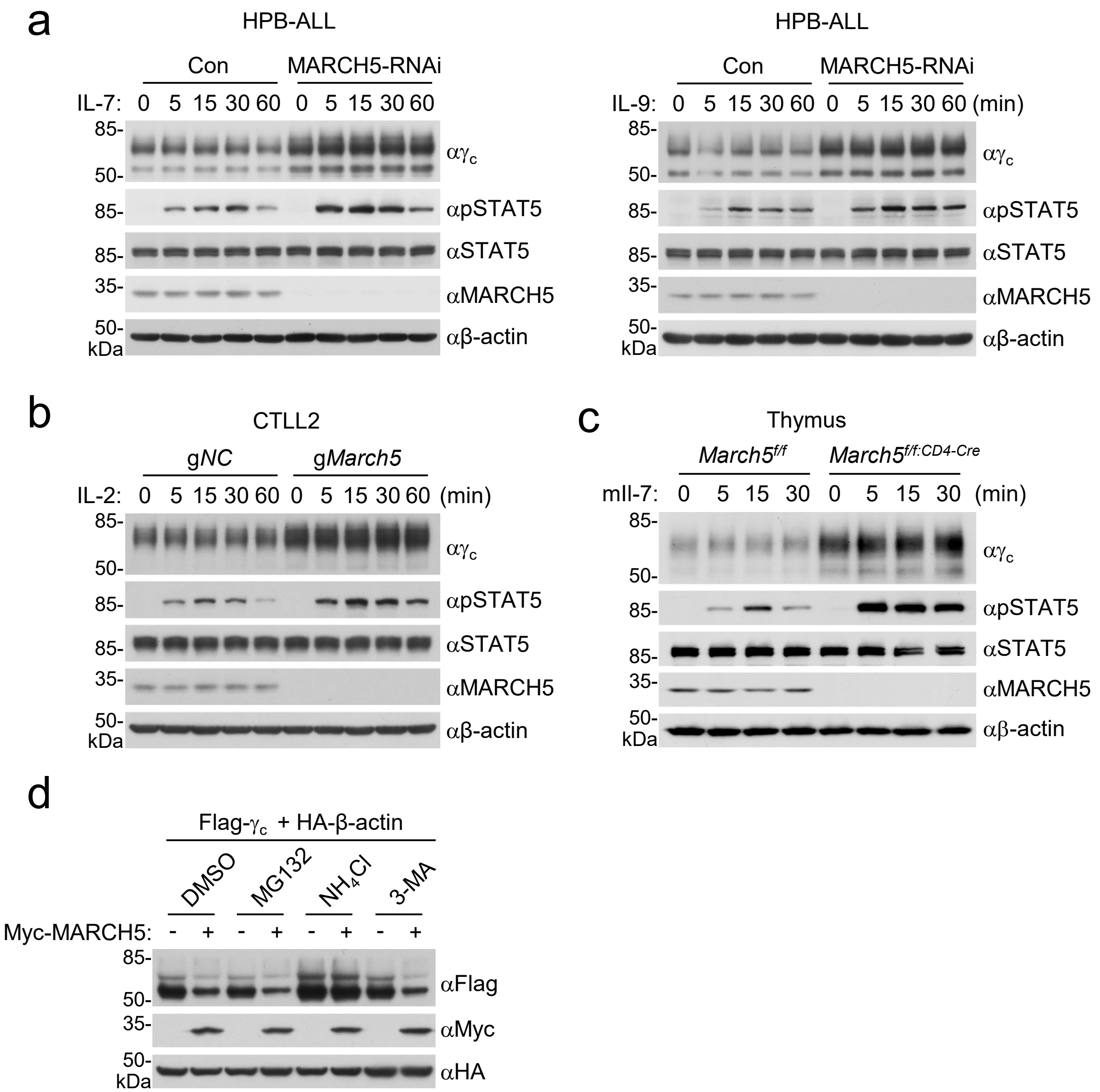

Supplementary information, Fig. S2 MARCH5 mediates K27-linked polyubiquitination and degradation of γ_c. Related to Fig. 2.

(a) MARCH5 knockdown up-regulates the protein level of γ_c and promotes γ_c family cytokine-triggered signaling. Control or MARCH5 knockdown HPB-ALL cells were stimulated with IL-7 (100 ng/mL) or IL-9 (100 ng/mL) for the indicated times before immunoblotting analysis with the indicated antibodies.

(b) MARCH5-deficiency up-regulates the level of γ_c and promotes IL-2-triggered signaling. Control or MARCH5-deficient CTLL2 cells were stimulated with IL-2 (400 IU/mL) for the indicated times before immunoblotting analysis with the indicated antibodies.

(c) MARCH5-deficiency up-regulates the protein level of γ_c and promotes Il-7-triggered signaling. Thymocytes from sex- and age-matched $March5^{f/f}$ or $March5^{f/f;CD4\text{-}Cre}$ mice were stimulated with mIl-7 (100 ng/mL) for the indicated times before immunoblotting analysis with the indicated antibodies.

(d) NH_4Cl inhibits MARCH5-induced degradation of γ_c. HEK293 cells were transfected with the indicated plasmids for 12 h, and then treated with MG132 (100 μM), NH_4Cl (25 mM) or 3-MA (500 ng/mL) for 6 h before immunoblotting analysis with the indicated antibodies.

All the experiments were repeated for at least two times with similar results
