# Supplementary material for: PD-1 signaling negatively regulates the common cytokine receptor γ chain via MARCH5-mediated ubiquitination and degradation to suppress anti-tumor immunity
Source: Cell Res. 2023 Nov 6;33(12):923–39. doi: 10.1038/s41422-023-00890-4 (PMC10709454; doi:10.1038/s41422-023-00890-4)
Supplement: Supplementary file 3 — Supplementary information, Fig. S3 [file 41422_2023_890_MOESM3_ESM.pdf]

Supplementary information, Fig. S3. Related to Fig. 2

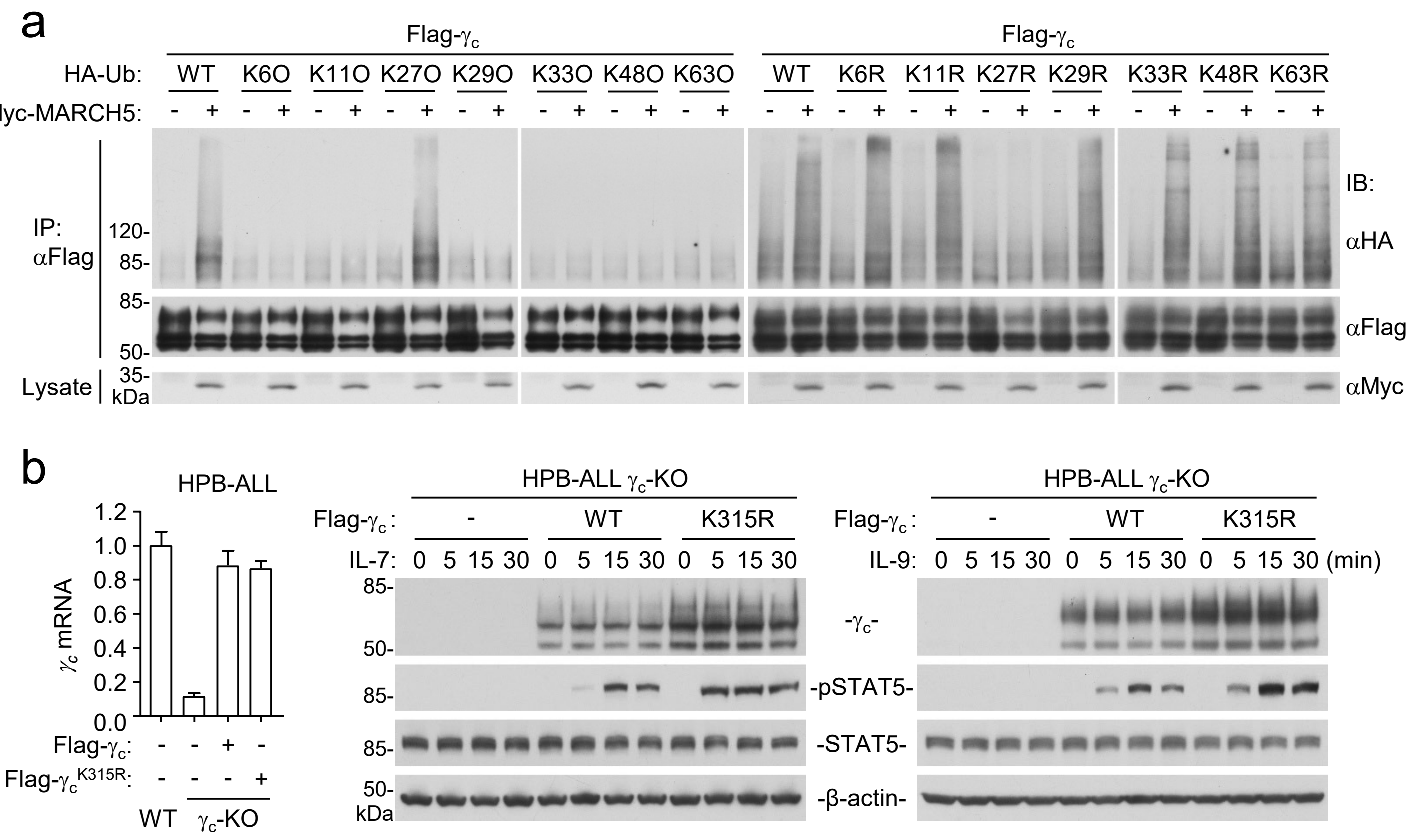

Supplementary information, Fig. S3 MARCH5 mediates K27-linked polyubiquitination and degradation of γ_c. Related to Fig. 2.

(a) MARCH5 promotes K27-linked polyubiquitination of γ_c. HEK293 cells were transfected with the indicated plasmids for 24 h before co-immunoprecipitation and immunoblotting analysis with the indicated antibodies.

(b) Effects of γ_c^{Y357F} on γ_c family cytokine-induced phosphorylation of $STAT5^{Y694/Y699}$. γ_c-deficient HPB-ALL cells were reconstituted with wild-type γ_c or γ_c^{K315R} before qPCR analysis of mRNA levels of the indicated genes (left panels). The cells were stimulated with IL-7 (100 ng/mL) or IL-9 (100 ng/mL) for the indicated times before immunoblotting analysis with the indicated antibodies (right panels).Graph shows mean ± SEM, $n = 3$ independent samples from one representative experiment. Data were analyzed using two-way ANOVA with GraphPad Prism 8.

All the experiments were repeated for at least two times with similar results.
