# Supplementary material for: PD-1 signaling negatively regulates the common cytokine receptor γ chain via MARCH5-mediated ubiquitination and degradation to suppress anti-tumor immunity
Source: Cell Res. 2023 Nov 6;33(12):923–39. doi: 10.1038/s41422-023-00890-4 (PMC10709454; doi:10.1038/s41422-023-00890-4)
Supplement: Supplementary file 4 — Supplementary information, Fig. S4 [file 41422_2023_890_MOESM4_ESM.pdf]

Supplementary information, Fig. S4. Related to Fig. 3

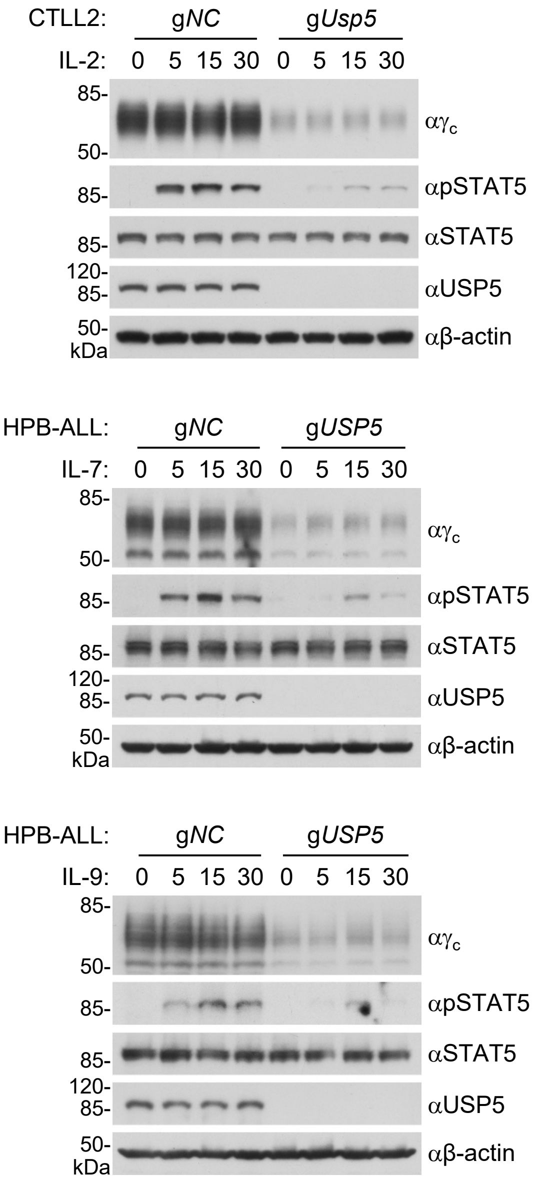

Supplementary information, Fig. S4 USP5-deficiency down-regulates γ_c level and inhibits the γ_c family cytokine-triggered signaling. Related to Fig. 3.

USP5-deficient (g*USP5*) or control (g*NC*) CTLL2 cells were stimulated with IL-2 (400 IU/mL), or USP5-deficient or control HPB-ALL cells were stimulated with IL-7 (100 ng/mL) or IL-9 (100 ng/mL) for the indicated times before immunoblotting analysis with the indicated antibodies. All the experiments were repeated for at least two times with similar results.
