# Supplementary material for: PD-1 signaling negatively regulates the common cytokine receptor γ chain via MARCH5-mediated ubiquitination and degradation to suppress anti-tumor immunity
Source: Cell Res. 2023 Nov 6;33(12):923–39. doi: 10.1038/s41422-023-00890-4 (PMC10709454; doi:10.1038/s41422-023-00890-4)
Supplement: Supplementary file 5 — Supplementary information, Fig. S5 [file 41422_2023_890_MOESM5_ESM.pdf]

Supplementary information, Fig. S5. Related to Fig. 5

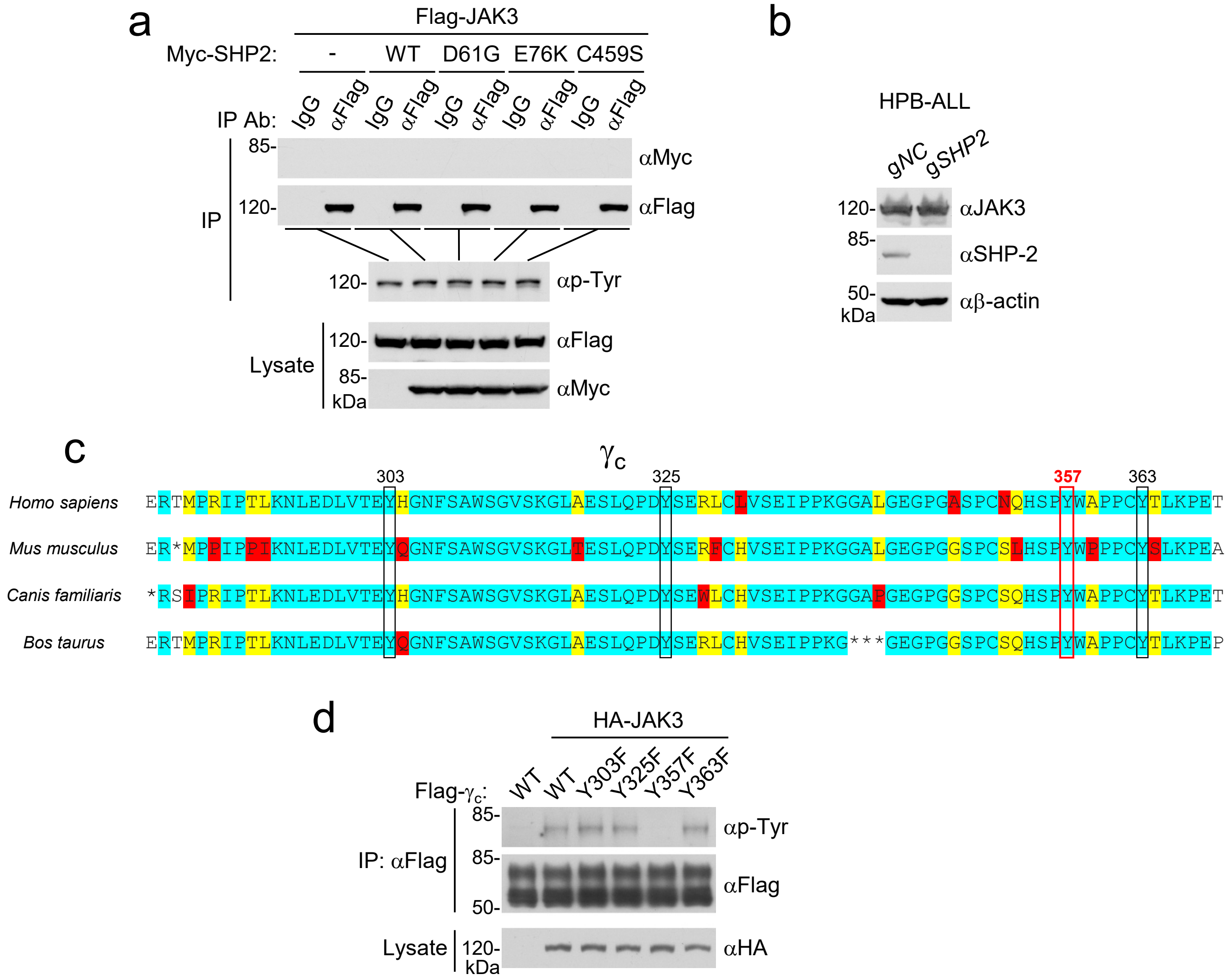

Supplementary information, Fig. S5 Mutation of γ_c^{Y357} to phenylalanine impairs its phosphorylation by JAK3. Related to Fig. 5.

(a) JAK3 is not associated with SHP2. HEK293 cells were transfected with the indicated plasmids for 24 h before co-immunoprecipitation and immunoblotting analysis with the indicated antibodies.

(b) Effects of SHP2-deficiency on JAK3 level. SHP2-deficient (g*SHP2*) or control (g*NC*) HPB-ALL cells were analyzed by immunoblots with the indicated antibodies.

(c) Sequence alignment of γ_c from the indicated species. The sequences are corresponding to aa284-369 of human γ_c.

(d) Mutation of γ_c^{Y357} to phenylalanine impairs its phosphorylation by JAK3. HEK293 cells were transfected with the indicated plasmids for 24 h before co-immunoprecipitation and immunoblotting analysis with the indicated antibodies.

All the experiments were repeated for at least two times with similar results.
