# Supplementary material for: PD-1 signaling negatively regulates the common cytokine receptor γ chain via MARCH5-mediated ubiquitination and degradation to suppress anti-tumor immunity
Source: Cell Res. 2023 Nov 6;33(12):923–39. doi: 10.1038/s41422-023-00890-4 (PMC10709454; doi:10.1038/s41422-023-00890-4)
Supplement: Supplementary file 6 — Supplementary information, Fig. S6 [file 41422_2023_890_MOESM6_ESM.pdf]

Supplementary information, Fig. S6. Related to Fig. 6

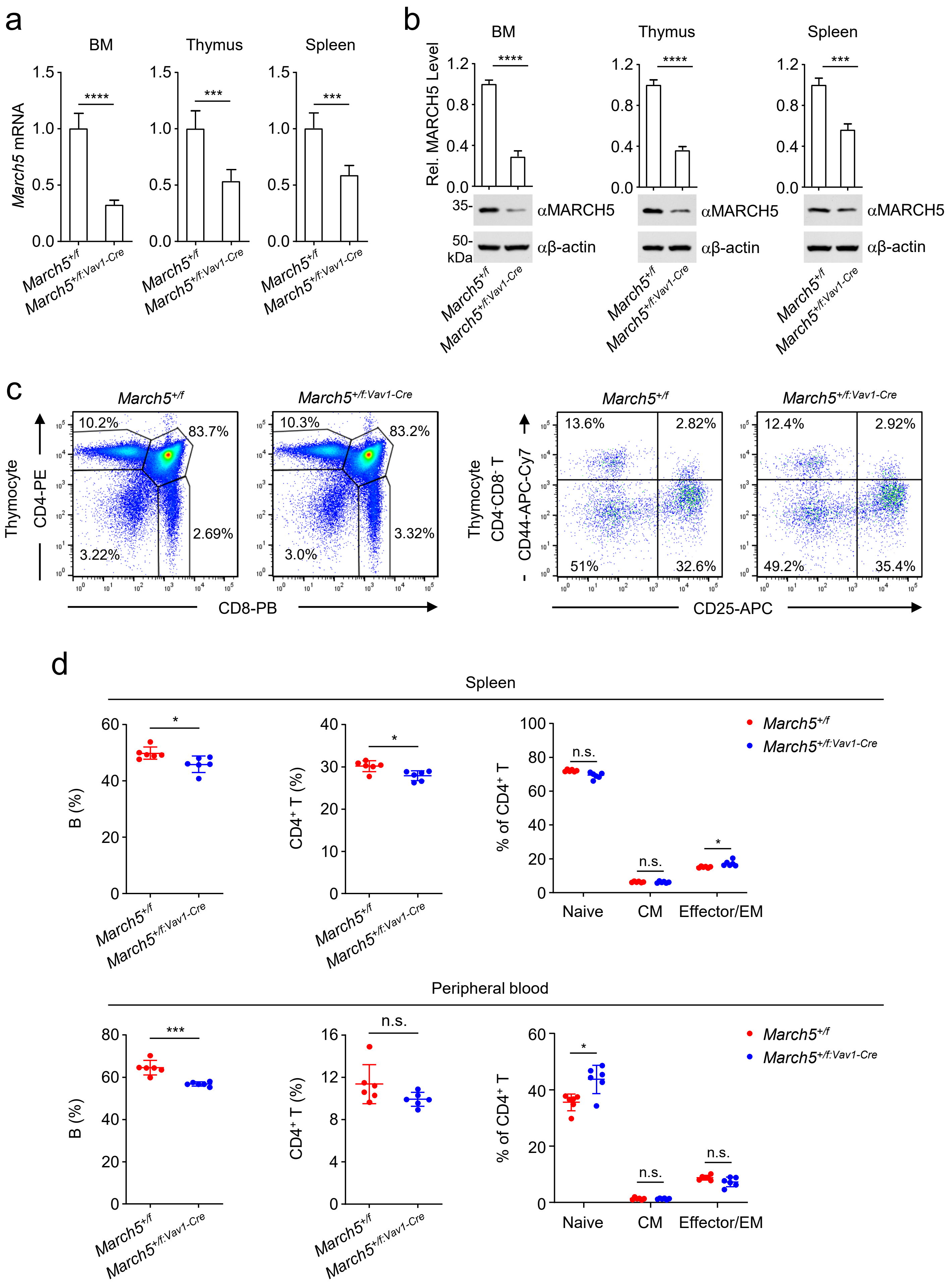

Supplementary information, Fig. S6 The percentages of various cell types from immune tissues of *March5*$^{+/f}$ and *March5*$^{+/f:Vav1\text{-}Cre}$ mice. Related to Fig. 6.

(a) The mRNA level of MARCH5 in immune tissues of *March5*$^{+/f}$ and *March5*$^{+/f:Vav1\text{-}Cre}$ mice. Cells (1×10^6) from bone marrow (BM), spleen and thymus were collected for qPCR analysis of MARCH5 mRNA level. Graph shows mean ± SEM, $n = 3$ independent samples from one representative experiment. Data were analyzed using a student's unpaired t-test with GraphPad Prism 8.

(b) MARCH5 protein level in immune tissues of *March5*$^{+/f}$ and *March5*$^{+/f:Vav1\text{-}Cre}$ mice. Cells (1×10^6) from bone marrow (BM), spleen and thymus were collected for immunoblotting analysis with the indicated antibodies. The MARCH5 band intensities relative to the corresponding β-actin bands were shown in the histograph. The immunoblots were repeated for three times with similar results. Graph shows mean ± SEM, $n = 3$ independent samples. Data were analyzed using a student's unpaired t-test with GraphPad Prism 8.

(c) The representative flow cytometric plots of thymocytes from *March5*$^{+/f}$ and *March5*$^{+/f:Vav1\text{-}Cre}$ mice. Thymocytes from sex- and age-matched *March5*$^{+/f}$ or *March5*$^{+/f:Vav1\text{-}Cre}$ mice were analyzed by flow cytometry for the percentage of $CD4^-CD8^-$ double-negative (DN), $CD4^+CD8^+$ double-positive (DP), $CD4^+$ single-positive (CD4SP), $CD8^+$ single-positive (CD8SP), $CD44^+$ single-positive of $CD4^-CD8^-$ double-negative cells (DN1), $CD44^+CD25^+$ double-positive of $CD4^-CD8^-$ double-negative cells (DN2), $CD25^+$ single-positive of $CD4^-CD8^-$ double-negative cells (DN3) and $CD44^-CD25^-$ double-negative of $CD4^-CD8^-$ double-negative cells (DN4).

(d) Effects of MARCH5 knockdown on the percentages of $CD4^+$ T and B cells from spleen and the peripheral blood. Splenocytes or peripheral blood leukocytes from sex- and age-matched *Marcf5*$^{+/f}$ or *Marcf5*$^{+/f:Vav1\text{-}Cre}$ mice were analyzed by flow cytometry for the percentage of $CD4^+$ T ($CD3^+CD4^+$), B ($CD3^-CD19^+$), native $CD4^+$ T ($CD44^{low}CD62L^{high}$ $CD4^+$ T cells), central memory $CD4^+$ T ($CD44^{high}CD62L^{high}$ $CD4^+$ T cells, CM) and effector/effector memory $CD4^+$ T cells ($CD44^{high}CD62L^{low}$ $CD4^+$ T cells, Effector/EM). Graph shows mean ± SEM, $n = 6$ independent samples. Data were analyzed using a student's unpaired t-test with GraphPad Prism 8.
