# Supplementary material for: PD-1 signaling negatively regulates the common cytokine receptor γ chain via MARCH5-mediated ubiquitination and degradation to suppress anti-tumor immunity
Source: Cell Res. 2023 Nov 6;33(12):923–39. doi: 10.1038/s41422-023-00890-4 (PMC10709454; doi:10.1038/s41422-023-00890-4)
Supplement: Supplementary file 7 — Supplementary information, Fig. S7 [file 41422_2023_890_MOESM7_ESM.pdf]

Supplementary information, Fig. S7. Related to Fig. 6

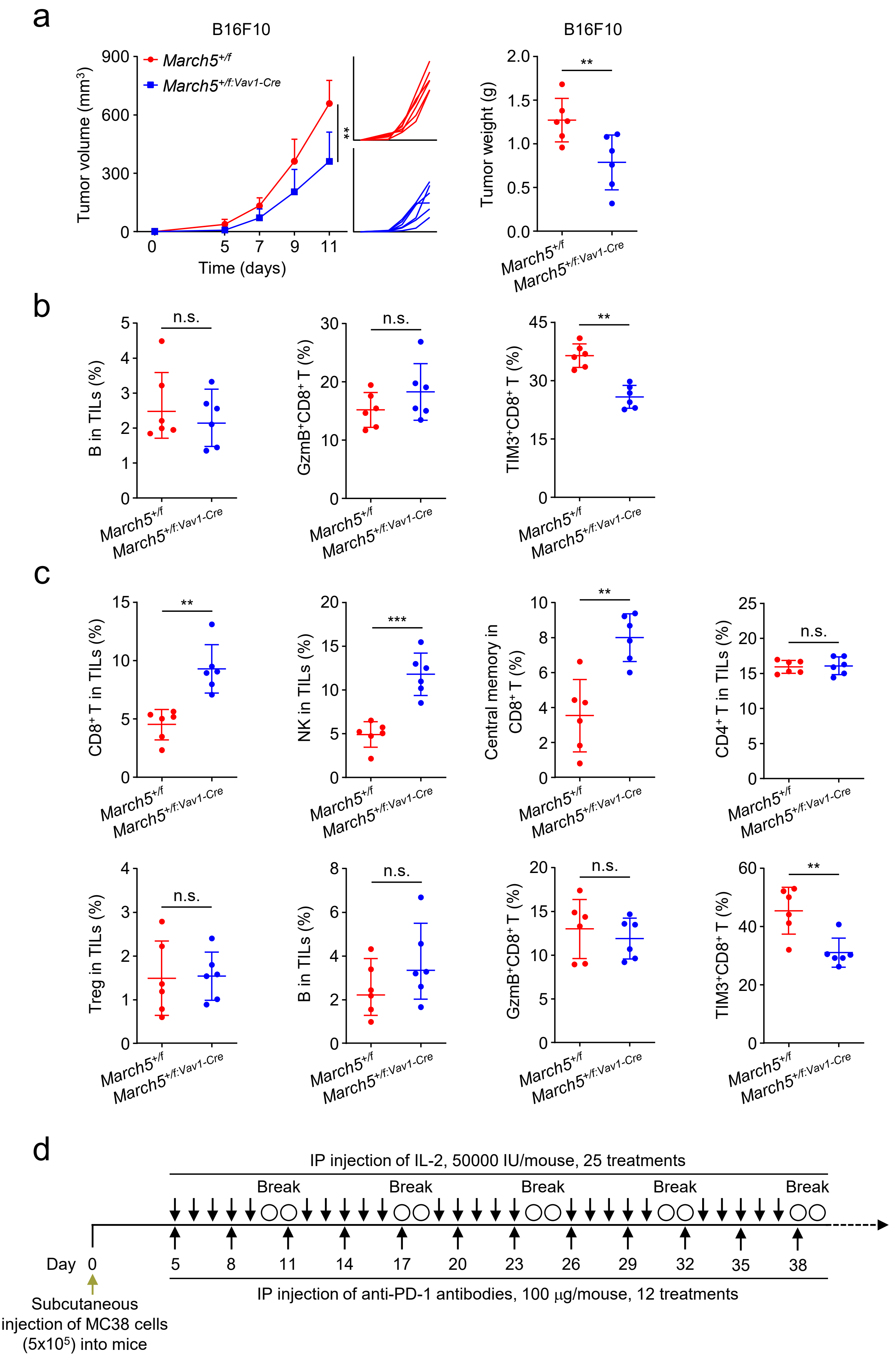

Supplementary information, Fig. S7 MARCH5 knockdown improves anti-tumor immunity and suppresses tumor growth. Related to Fig. 6.

(a) MARCH5 knockdown inhibits tumor growth. Sex- and age-matched *March5*$^{+/f}$ or *March5*$^{+/f:Vav1\text{-}Cre}$ mice were subcutaneously injected with B16F10 cells (5×10^5). On day 5 after tumor cell implantation, tumor sizes were measured every two days by caliper. Tumour-bearing mice were euthanized on day 13. Tumor weights were measured by Analytical Balance. Graph shows mean ± SEM, *n* = 6. Data were analyzed using a student's unpaired t-test with GraphPad Prism 8.

(b) Effects of MARCH5 knockdown on the percentages of various cell types from TILs. TILs were isolated from the MC38 tumor tissues of Fig. 6d. TILs were stained with the indicated antibodies and analyzed by flow cytometry. Graph shows mean ± SEM, *n* = 6 independent samples. Data were analyzed using a student's unpaired t-test with GraphPad Prism 8.

(c) Effects of MARCH5 knockdown on the percentages of various cell types from TILs. TILs were isolated from the B16F10 tumor tissues of Fig. S7a. TILs were stained with the indicated antibodies and analyzed by flow cytometry. Graph shows mean ± SEM, *n* = 6 independent samples. Data were analyzed using a student's unpaired t-test with GraphPad Prism 8.

(d) A schematic treatment plan for *March5*$^{+/f}$ and *March5*$^{+/f:Vav1\text{-}Cre}$ mice bearing subcutaneous MC38 tumors. Related to Fig. 6f, g.
