# Supplementary material for: PD-1 signaling negatively regulates the common cytokine receptor γ chain via MARCH5-mediated ubiquitination and degradation to suppress anti-tumor immunity
Source: Cell Res. 2023 Nov 6;33(12):923–39. doi: 10.1038/s41422-023-00890-4 (PMC10709454; doi:10.1038/s41422-023-00890-4)
Supplement: Supplementary file 8 — Supplementary information, Fig. S8 [file 41422_2023_890_MOESM8_ESM.pdf]

Supplementary information, Fig. S8. Related to Fig. 6

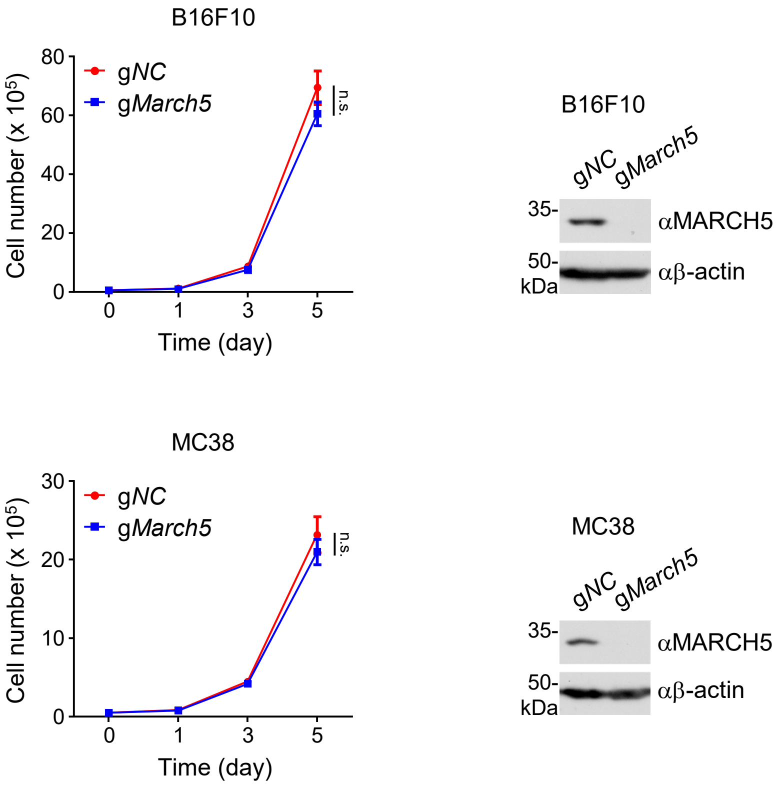

Supplementary information, Fig. S8 MARCH5-deficiency has no significant effects on the proliferation of B16F10 or MC38 cells . Related to Fig. 6.

MARCH5-deficient (g*March5*) and control (g*NC*) B16F10 or MC38 cells were subjected to proliferation analysis. Lysates of the indicated cells were analyzed by immunoblots with the indicated antibodies. Graph shows mean ± SEM, $n = 3$. Data were analyzed using a student's unpaired t-test with GraphPad Prism 8.
