# Supplementary material for: PD-1 signaling negatively regulates the common cytokine receptor γ chain via MARCH5-mediated ubiquitination and degradation to suppress anti-tumor immunity
Source: Cell Res. 2023 Nov 6;33(12):923–39. doi: 10.1038/s41422-023-00890-4 (PMC10709454; doi:10.1038/s41422-023-00890-4)
Supplement: Supplementary file 9 — Supplementary information, Fig. S9 [file 41422_2023_890_MOESM9_ESM.pdf]

Supplementary information, Fig. S9. Related to Fig. 7

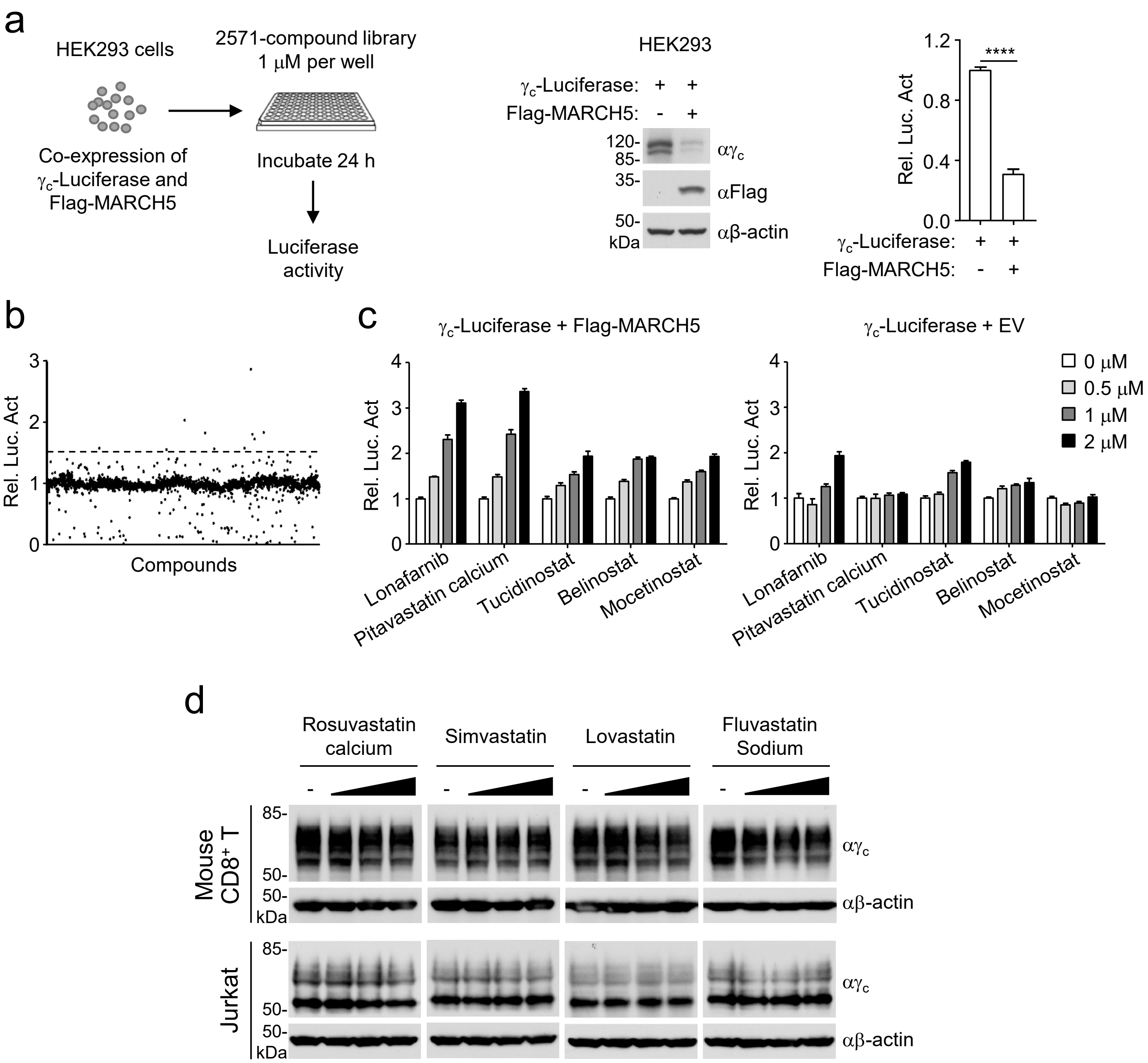

Supplementary information, Fig. S9 PC negatively regulates γ_c level. Related to Fig. 7.

(a) Schematic of chemical screens. HEK293 cells were co-transfected with two plasmids expressing MARCH5 and γ_c-luciferase fusion protein respectively. A total of 2571 of Food and Drug Administration-approved drugs (1 μM of each compound) were used in the screens. The γ_c-luciferase expressing or γ_c-luciferase + MARCH5 co-expressing HEK293 cells were collected for immunoblotting analysis and luciferase reporter assays. Graph shows mean ± SEM, $n = 3$ independent samples. Data were analyzed using a student's unpaired t-test with GraphPad Prism 8.

(b) Scatter plots showing the relative inhibitory effects of chemicals in MARCH5-mediated γ_c degradation.

(c) Effects of top 5 compounds on γ_c-luciferase activity. MARCH5-expressing or control HEK293 cells were treated with the indicated compounds (0, 0.5, 1, 2 μM) for 24 h before luciferase reporter assays. Graph shows mean ± SEM, $n = 3$ independent samples.

(d) Effects of HMG-CoA reductase inhibitors on γ_c level. Mouse $CD8^+$ T or Jurkat cells were treated with HMG-CoA reductase inhibitors (Rosuvastatin calcium, Simvastatin, Lovastatin or Fluvastatin Sodium) (0, 0.5, 1, 2 μM) for 24 h before immunoblotting analysis with the indicated antibodies. The immunoblots were repeated for two times with similar results.
