# Supplementary material for: PD-1 signaling negatively regulates the common cytokine receptor γ chain via MARCH5-mediated ubiquitination and degradation to suppress anti-tumor immunity
Source: Cell Res. 2023 Nov 6;33(12):923–39. doi: 10.1038/s41422-023-00890-4 (PMC10709454; doi:10.1038/s41422-023-00890-4)
Supplement: Supplementary file 10 — Supplementary information, Fig. S10 [file 41422_2023_890_MOESM10_ESM.pdf]

Supplementary information, Fig. S10. Related to Fig. 7

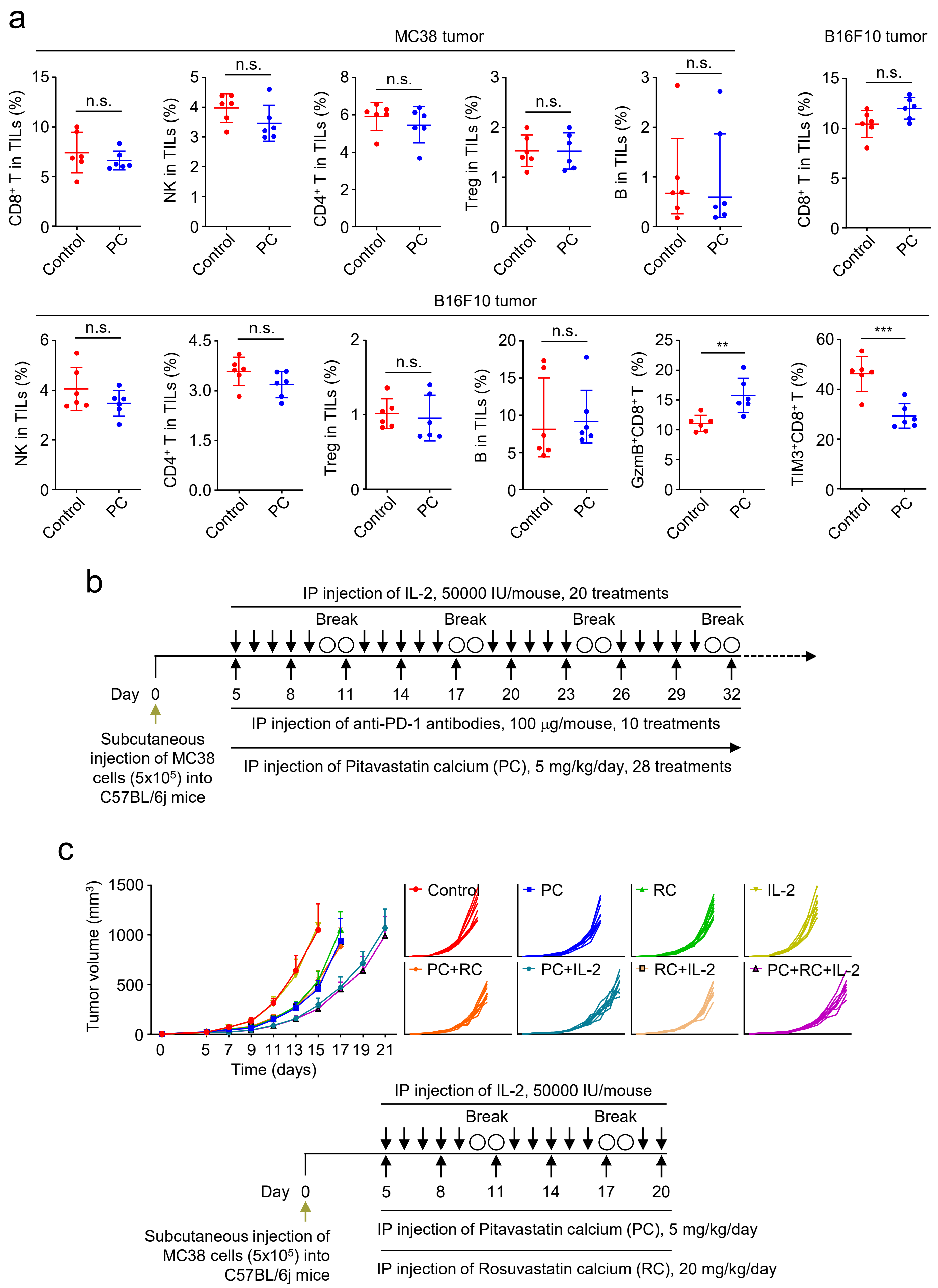

**Supplementary information, Fig. S10 PC promotes anti-tumor immunity. Related to Fig. 7.**

**(a)** Effects of PC on the percentages of various cell types from TILs. TILs were isolated from the MC38 or B16F10 tumor tissues of Fig. 7c. TILs were stained with the indicated antibodies and analyzed by flow cytometry. Graph shows mean ± SEM, $n = 6$ independent samples. Data were analyzed using a student's unpaired t-test with GraphPad Prism 8.

**(b)** A schematic treatment plan for C57BL/6j mice bearing subcutaneous MC38 tumors. Related to Fig. 7f, g.

**(c)** PC but not RC potentiates the anti-tumor efficacy of IL-2. C57BL/6j mice were subcutaneously injected with MC38 cells ($5\times10^5$). On day 5 after tumor cell implantation, mice were intraperitoneally injected with control, PC (5 mg/kg), RC (20 mg/kg) or IL-2 (50000 IU per mouse as indicated). Tumor sizes were measured every two days by caliper from day 5. Graph shows mean ± SEM, $n = 8$. Data were analyzed using two-way ANOVA with GraphPad Prism 8.
