# Supplementary material for: PD-1 signaling negatively regulates the common cytokine receptor γ chain via MARCH5-mediated ubiquitination and degradation to suppress anti-tumor immunity
Source: Cell Res. 2023 Nov 6;33(12):923–39. doi: 10.1038/s41422-023-00890-4 (PMC10709454; doi:10.1038/s41422-023-00890-4)
Supplement: Supplementary file 12 — Supplementary information, Table S2 [file 41422_2023_890_MOESM12_ESM.pdf]

Supplementary information, Table S2. A list of compound leads exhibiting more than 1.5-fold effects in reversal of γ_c-luciferase activity

Rank	Drug	Rel. Luc. Act	Target
1	Lonafarnib	2.88	Raf/Ras
2	Pitavastatin calcium	2.06	HMG-CoA reductase
3	Tucidinostat	1.86	HDAC1/2/3/10
4	Belinostat	1.85	HDAC
5	Mocetinostat	1.83	HDAC1/2/3/11
6	Pracinostat	1.75	HDAC
7	Risedronate Sodium	1.60	HBV
8	Entinostat	1.60	HDAC1/3
9	Chidamide	1.59	HDAC1/2/3/8
10	Vorinostat	1.58	HDAC1/2/3/6/8
