# Supplementary material for: PD-1 signaling negatively regulates the common cytokine receptor γ chain via MARCH5-mediated ubiquitination and degradation to suppress anti-tumor immunity
Source: Cell Res. 2023 Nov 6;33(12):923–39. doi: 10.1038/s41422-023-00890-4 (PMC10709454; doi:10.1038/s41422-023-00890-4)
Supplement: Supplementary file 13 — Supplementary information, Table S3 [file 41422_2023_890_MOESM13_ESM.pdf]

Supplementary information, Table S3. A list of primary antibodies used in the study

**Antibody**	**Supplier**	**Catalog No.**	**Appl.[a]**	**Usage**
Mouse anti-Flag M2 antibody clone M2	Sigma-Aldrich	#F3165	WB/IP	1:2000/1 μg
Mouse anti-HA. 11 Epitope Tag antibody clone 16B12	BioLegend	#901515	WB/IP	1:2000/1 μg
Mouse anti-Myc-Tag antibody clone 9B11	Cell Signaling Technology	#2276S	WB	1:2000
Mouse anti-β-actin antibody clone AC-74	Sigma-Aldrich	#A2228	WB	1:5000
Rabbit anti-γ_c polyclonal antibody	Proteintech	#11409-1-AP	IP	2 μg
Rabbit anti-γ_c antibody clone EPR24694-133	Abcam	#ab273023	WB/IHC	1:1000/1:500
Rabbit anti-Ubiquitin antibody clone EPR8830	Abcam	#ab134953	WB	1:1000
Rabbit anti-IL2Rβ antibody clone D4X3H	Cell Signaling Technology	#46307S	WB	1:1000
Rabbit anti-MARCH5 antibody	Abcam	#ab77585 #ab185054	WB	1:1000
Rabbit anti-MARCH5 antibody	Cell Signaling Technology	#19168S	WB	1:1000
Rabbit anti-Ubiquitin (linkage-specific K27) antibody clone EPR17034	Abcam	#ab181537	WB	1:1000
Rabbit anti-USP5 polyclonal antibody	Proteintech	#10473-1-AP	WB	1:1000
Mouse anti-BATF antibody clone WW8	Santa Cruz Biotechnology	#sc-100974	WB/CHIP	1:500/2 μg
Rabbit anti-SHP2 antibody clone D50F2	Cell Signaling Technology	#3397S	WB	1:1000
Mouse anti-Phosphor-Tyrosine antibody clone P-Tyr-100	Cell Signaling Technology	#9411S	WB	1:1000

Rabbit anti-STAT5 antibody clone D2O6Y	Cell Signaling Technology	94205	WB	1:2000
Rabbit anti-pSTAT5 (Tyr694) antibody clone D47E7	Cell Signaling Technology	#4322	WB	1:1000
Rabbit anti-PD-L1 antibody clone E1L3N	Cell Signaling Technology	#13684	IHC	1:200
Rabbit anti-PD-1 antibody clone D4W2J	Cell Signaling Technology	#86163	WB	1:1000
Rabbit anti-JAK3 antibody clone D1H3	Cell Signaling Technology	#8827	WB	1:1000
