# Supplementary material for: PD-1 signaling negatively regulates the common cytokine receptor γ chain via MARCH5-mediated ubiquitination and degradation to suppress anti-tumor immunity
Source: Cell Res. 2023 Nov 6;33(12):923–39. doi: 10.1038/s41422-023-00890-4 (PMC10709454; doi:10.1038/s41422-023-00890-4)
Supplement: Supplementary file 14 — Supplementary information, Table S4 [file 41422_2023_890_MOESM14_ESM.pdf]

Supplementary information, Table S4. A list of gRNA sequences

Human MARCH5	5’-ATCCACCCAGCGTTGTAGAC-3’
Human USP5	5’-CGTGTTCATACAGATGTAGAGG-3’
Human BATF	5’-GCGAGCGACATGTCCCTTTG-3’
Human IL2RG	5’-TCTTCAGGGTGGGAATTCGGGG-3’
Human SHP2	5’-GTGATTACTATGACCTGTATGG-3’
Mouse March5	5’-GGTTCCTGCCGGTCCGCCCA-3’
Mouse Usp5	5’-CGCTCATTGGATGCGAGAGA-3’
Mouse Shp2	5’-CTCCGCGGGGTACCGTCACA-3’
