# Supplementary material for: PD-1 signaling negatively regulates the common cytokine receptor γ chain via MARCH5-mediated ubiquitination and degradation to suppress anti-tumor immunity
Source: Cell Res. 2023 Nov 6;33(12):923–39. doi: 10.1038/s41422-023-00890-4 (PMC10709454; doi:10.1038/s41422-023-00890-4)
Supplement: Supplementary file 15 — Supplementary information, Table S5 [file 41422_2023_890_MOESM15_ESM.pdf]

Supplementary information, Table S5. A list of qPCR sequences

Human GAPDH	GTCTCCTCTGACTTCAACAGCG
	ACCACCCTGTTGCTGTAGCCAA
Human IL2RG	CACTCTGTGGAAGTGCTCAGCA
	GAGCCAACAGAGATAACCACGG
Human MARCH5	AGAGTGGCATGTCCTCAGTGCA
	AGCCGACCATTATTCCTGCTGC
Mouse Gapdh	ACGGCCGCATCTTCTTGTGCA
	ACGGCCAAATCCGTTCACACC
Mouse March5	ACCATGCAGGTGCAGAGGATC
	AAACACTATTAAGTACTCGGCGTT
