# Supplementary material for: PD-1 signaling negatively regulates the common cytokine receptor γ chain via MARCH5-mediated ubiquitination and degradation to suppress anti-tumor immunity
Source: Cell Res. 2023 Nov 6;33(12):923–39. doi: 10.1038/s41422-023-00890-4 (PMC10709454; doi:10.1038/s41422-023-00890-4)
Supplement: Supplementary file 16 — Supplementary information, Table S6 [file 41422_2023_890_MOESM16_ESM.pdf]

Supplementary information, Table S6. A list of qPCR sequences for CHIP analysis

Human MARCH5 #1	AGTGGAGACGGTCCGAAGA
	TGTGAGGTAAGAGTCGCCG
Human MARCH5 #2	TGGAGACGGTCCGAAGACTA
	GCGGAGGAGCTACCTTTGTG
