# Supplementary material for: PD-1 signaling negatively regulates the common cytokine receptor γ chain via MARCH5-mediated ubiquitination and degradation to suppress anti-tumor immunity
Source: Cell Res. 2023 Nov 6;33(12):923–39. doi: 10.1038/s41422-023-00890-4 (PMC10709454; doi:10.1038/s41422-023-00890-4)
Supplement: Supplementary file 17 — Supplementary information, Table S7 [file 41422_2023_890_MOESM17_ESM.pdf]

Supplementary information, Table S7. A list of flow antibodies used in the study

Antibody	Clone	Supplier	Catalog No.
PE anti-human CD132 antibody	TUGh4	BioLegend	#338606
APC anti-human CD3 antibody	OKT3	BioLegend	#317318
FITC anti-human CD8 antibody	RPA-T8	BD Biosciences	#557085
APC anti-mouse CD132 antibody	TUGm2	BioLegend	#132308
PE-Cy7 anti-mouse CD3 antibody	145-2C11	BD Biosciences	#552774
PE anti-mouse CD4 antibody	RM4-5	BD Biosciences	#553048
PB anti-mouse CD8 antibody	53-6.7	BD Biosciences	#558106
APC-Cy7 anti-mouse CD44 antibody	IM7	BioLegend	#103028
APC anti-mouse CD25 antibody	PC61	BioLegend	#102012
PerCP-Cy5.5 anti-mouse CD4 antibody	RM4-5	Invitrogen	#45-0042-82
FITC anti-mouse NKp46 antibody	29A1.4	BioLegend	#137606
PE anti-mouse CD19 antibody	1D3	BD Biosciences	#557399
APC anti-mouse CD62L antibody	MEL-14	BioLegend	104412
PerCP-710 anti-mouse GzmB antibody	NGZB	Invitrogen	#46-8898-82
PE anti-mouse TIM3 antibody	RMT3-23	Invitrogen	#12-5870-82
APC anti-mouse CD69 antibody	H1.2F3	Invitrogen	#17-0691-82
APC anti-mouse Foxp3 antibody	FJK-16s	Invitrogen	#17-5773-82
